# Biomarker profiles in serum and saliva of experimental Sjögren's syndrome: associations with specific autoimmune manifestations

**DOI:** 10.1186/ar2375

**Published:** 2008-02-20

**Authors:** Nicolas Delaleu, Heike Immervoll, Janet Cornelius, Roland Jonsson

**Affiliations:** 1 Broegelmann Research Laboratory, The Gade Institute, University of Bergen, Haukelandsveien, Bergen 5021, Norway; 2 Section of Pathology, The Gade Institute, University of Bergen, Jonas Liesvei, Bergen 5021, Norway; 3 Department of Pathology, Haukeland University Hospital, Jonas Liesvei, Bergen 5021, Norway; 4 Department of Pathology, Immunology and Laboratory Medicine, University of Florida, SW Archer Road, Gainesville, FL 32610, USA; 5 Department of Rheumatology, Haukeland University Hospital, Bergen, Jonas Liesvei, Bergen 5021, Norway; 6 Department of Otolaryngology, Head and Neck Surgery, Haukeland University Hospital, Bergen, Jonas Liesvei, Bergen 5021, Norway

## Abstract

**Introduction:**

Sjögren's syndrome (SS) is a systemic autoimmune disease that mainly targets the exocrine glands. The aim of this study was to investigate the involvement of 87 proteins measured in serum and 75 proteins analyzed in saliva in spontaneous experimental SS. In addition, we intended to compute a model of the immunological situation representing the overt disease stage of SS.

**Methods:**

Nondiabetic, nonobese diabetic (NOD) mice aged 21 weeks were evaluated for salivary gland function, salivary gland inflammation and extraglandular disease manifestations. The analytes, comprising chemokines, cytokines, growth factors, autoantibodies and other biomarkers, were quantified using multi-analyte profile technology and fluorescence-activated cell sorting. Age-matched and sex-matched Balb/c mice served as a reference.

**Results:**

We found NOD mice to exhibit impaired salivary flow, glandular inflammation and increased secretory SSB (anti-La) levels. Thirty-eight biomarkers in serum and 34 in saliva obtained from NOD mice were significantly different from those in Balb/c mice. Eighteen biomarkers in serum and three chemokines measured in saliva could predict strain membership with 80% to 100% accuracy. Factor analyses identified principal components mostly correlating with one clinical aspect of SS and having distinct associations with components extracted from other families of proteins.

**Conclusion:**

Autoimmune manifestations of SS are greatly independent and associated with various immunological processes. However, CD40, CD40 ligand, IL-18, granulocyte chemotactic protein-2 and anti-muscarinic M3 receptor IgG_3 _may connect the different aspects of SS. Processes related to the adaptive immune system appear to promote SS with a strong involvement of T-helper-2 related proteins in hyposalivation. This approach further established saliva as an attractive biofluid for biomarker analyses in SS and provides a basis for the comparison and selection of potential drug targets and diagnostic markers.

## Introduction

Over recent decades the immune system has been subject to much investigation. Growing complexity has often been a major byproduct of the discoveries reported, and subsequently models were established to cope with such complexity. Regarding autoimmune diseases in general, and Sjögren's syndrome (SS) in particular, verifying and expanding such models is desirable, because it has proved difficult to extrapolate findings to existing models that were often developed in different contexts [1-3]. Recent technological advances have greatly increased the amount of information and the number of proteins that can be investigated in any given system and put into a scientific context simultaneously. These technologies, termed transcriptomics, proteomics, metabolomics and other '-omics', were followed by the an increase in systems-based thinking across different scientific disciplines [4]. This trend has promoted systems biology from a technology-driven enterprise to an innovative tool in drug discovery, and it may lead to a more complete perspective on how specific components contribute at different system levels to the immune response. Contextualization and integration have been key drivers of such approaches.

SS (for review [5,6]), a systemic autoimmune disease, is manifested by severe impairment of exocrine gland function and focal mononuclear cell infiltrates within the salivary and lacrimal glands. The identification of anti-M3 receptor (M3R) autoantibodies for the first time attributed a defined pathogenic role to an autoantibody in SS [7-9]. The roles of other autoantibodies in the pathogenesis of SS (especially SSA [anti-Ro] and SSB [anti-La], which are frequently present in patients with SS) remain to be determined. The disease can involve organs other than the exocrine glands, and the worst disease outcome – lymphoid malignancy – develops in up to 5% of patients with SS. Currently, applied treatments provide merely marginal symptomatic relief [10,11].

The nonobese diabetic (NOD) mouse, which spontaneously develops both SS-like histopathology and hyposalivation, is the most widely accepted model for SS [12,13]. Based on the findings of studies conducted in these mice [14], it is thought that the various SS-related manifestations develop according to a specific time course. However, similar to human SS, the immunological relationship between the two hallmarks of SS, namely salivary gland inflammation and hyposalivation, is far from being understood in NOD mice. Although some diabetes-related genetic loci might contribute to the SS-like disease in NOD mice, both autoimmune diseases can develop independently from each other [15]. Onset of SS in NOD mice is not critically dependent on the diabetes-related H2g7 haplotype. NOD.B10.H2b congenic mice also exhibit an SS-like disease in the absence of overt diabetes [16], which prevents exclusion of diabetic animals from studies conducted in SS. However, similar to nondiabetic parental NOD mice, they exhibit lymphoid infiltration in the pancreas and, rarely, insulitis [17]. Another model of SS, the C57BL/6.NOD-Aec1Aec2 strain, has not been screened for SS-unrelated autoimmune manifestations other than insulitis [15]. However, the background strain C57BL/6 develops spontaneous organ-specific autoimmune lesions in salivary glands, pancreas, kidneys, lungs and liver, and produces a variety of autoantibodies [18].

The analytes investigated in this study, which were previously studied in humans or NOD mice within the context of SS, are listed in Additional file 1 (Supplementary table 1). Few studies have assessed interactions between several immune molecules and their association with disease parameters.

Summarizing findings regarding immune mediators such as cytokines in SS, it was concluded that T-helper (Th)2 cytokines are predominant in an early phase of SS, whereas Th1 cytokines are associated with a later stage of the disease [19]. In opposition stands the proposed principle that decreased salivary flow, potentially associated with Th2 cytokines, follows the emergence of glandular inflammation, which was linked to a Th1 response [14,20]. In addition, the transition between the preclinical and the overt disease state has been associated with shifts in cytokine profiles in NOD mice [14] and the IL-4/signal transducer and activator of transcription (STAT)6 pathway [20]. Chemokines, small secreted proteins, have been implicated in leucocyte chemoattraction, angiogenesis, fibrosis and malignancy [21]. Despite their uncontested potential as targets for therapeutic intervention, few studies have examined chemokines within the context of SS (Additional file 1 [Supplementary table 1]).

The purpose of the present study was to expand knowledge regarding 87 analytes in serum and 75 proteins in saliva. Thirty and 62 of these molecules have not yet been investigated in SS patients and SS-like disease in NOD mice, respectively. Thirty-six and 54 of the biomarkers have not yet been analyzed in serum and saliva obtained from patients with SS, and neither have 70 of the analytes in serum and 62 biomarkers in saliva from NOD mice been evaluated in a SS-specific context. Based on direct comparison with Balb/c mice, we intended to identify differentially expressed proteins and investigate their potential to discriminate between the disease model and the control strain. This pool of data should also allow computation of a correlation network, representing associations of biomarkers with relevant clinical features of SS in nondiabetic NOD mice, both systemically and locally.

## Materials and methods

### Animals and assessment of diabetes

Twenty-two female NOD/LtJ (stock #001976) and 19 female Balb/cJ (stock #000651) mice (The Jackson Laboratory, Bar Harbor, ME, USA) were housed in individually ventilated cages at the animal facility of the Department of Physiology, University of Bergen, Bergen, Norway. The study was approved by the Committee for Research on Animals/Forsøksdyrutvalget (project #12-05/BBB).

To serve as controls for subsequent immunostimulatory intervention studies, all mice were injected subcutaneously at 7 weeks of age with 25 μl incomplete Freund's adjuvant emulsified in phosphate-buffered saline. From 10 weeks onward NOD mice were screened weekly for diabetes. Two repeated measurements of glucosuria (>50 mg/dl; Keto-Diabur-Test strips, Roche, Mannheim, Germany) were considered to represent onset of diabetes. At weeks 20 and 21, all mice were screened for hyperglycaemia (>300 mg/dl; Ascensia-microfill, Bayer Healthcare, Mishawaka, IN, USA). At 21 weeks, 10 out of 22 mice (45.5%) were considered to be diabetic and were excluded from all subsequent analyses. We excluded these animals in order to eliminate from our findings any SS-unrelated impact of hyperglycaemia on the physiological process of saliva secretion and the anticipated distorting effect of hyperglycaemia on biomarker profiles.

### Measurement of stimulated salivary flow

Mice were fasted but given water *ad libitum *and anaesthetized with an intramuscular injection of ketamine and medetomidine. Salivary secretion was induced by intraperitoneal injection of 0.5 μg pilocarpine/g body weight (#P6503; Sigma, St. Louis, MO, USA) and collected during 10 minutes. Pre-weighed tubes were weighed again after collection to determine the amount of saliva (1 μg = 1 μl). Protease inhibitor cocktail (#P8340; Sigma) was added at a concentration of 1:500 and samples were kept at -80°C until analysis.

### Blood sampling and organ collection

Blood was collected from the saphenous vein from nonanaesthetized mice and by heart puncture on the day of euthanasia. The blood was allowed to clot and centrifuged for 10 minutes at 800 *g *to obtain serum. The organs were fixed in 4% formalin before embedding in paraffin, sectioning, and staining with haematoxylin and eosin. Sections obtained from the kidneys were also stained using the periodic acid-Schiff staining technique.

### Evaluation of salivary gland inflammation and insulitis in the pancreas

After qualitative evaluation of three independent sections, the section with the highest degree of inflammation was recorded as a whole, creating a multiple image-composite picture. A graph tablet was used to select and morphometrically measure the total glandular area and the individual size of each focus. Subsequently, focus score (FS; number of foci of 50 or more mononuclear cells/mm^2 ^glandular tissue) and ratio index (RI; area of inflammation/area of glandular tissue) were determined.

To determine the insulitis score (IS), at least five haematoxylin and eosin stained tissue sections of the pancreas were analyzed in a blinded manner. On average, 32 islets per mouse were scored, as described by Leiter [22].

### Multi-analyte profiles from serum and saliva

A bead-based multiplex sandwich immunofluorescence assay was used to generate multi-analyte profiles (MAPs) from serum and saliva from the 12 nondiabetic NOD and 12 Balb/c mice, comprising 82 analytes for serum and 75 for saliva (Additional file 1 [Supplementary table 2]). Analyses were conducted at Rules Based Medicine Inc. (Austin, TX, USA) using a fully automated system. For each multiplex, eight-point calibrators and three-level controls were included on each microtitre plate. Antibodies used in the MAP to recognize and quantify the specific autoantibodies were directed against all isotypes.

### Quantification of anti-M3R antibodies

Levels of anti-M3R autoantibodies were measured as described previously [23]. In brief, aliquots of 2 × 10^5 ^Chinese hamster ovary cells, transfected with pcDNA5/FRT/V5-His MsM3R-Flp-In cells, were incubated for 1.5 hours at 4°C with 10 μl of serum before incubation with one of the following fluorescein isothiocyanate (FITC)-conjugated goat anti-mouse detection antibodies (purchased from Southern Biotech) diluted 1:50: isotype control, goat IgG (#0110-02); IgG (H+L; #1031-02); IgG_1 _F(ab')2 (#1072-02); IgG_2b _F(ab')2 (#1092-02); IgG_2c _F(ab')2 (#1079-02); and IgG_3 _F(ab')2 (#1102-02). The cells were analyzed using a FACSCalibur flow cytometer using Cell Quest software (BD Biosciences, San Jose, CA, USA) and FlowJo (Tree Star Inc., Ashland, OR, USA). The quantities of anti-M3R autoantibodies were analyzed by gating on the FITC-positive population situated above the threshold, set by the sample stained with the secondary antibody alone. The percentage of positive cells was calculated to represent the quantity of anti-M3R autoantibodies.

### Statistical analyses

Means were compared using independent Student's *t*-test (two-tailed). Bivariate linear associations, used to generate the correlation matrixes, were computed using two-tailed Pearson correlation (r). Strain membership prediction was assessed by discriminant analyses (DA) and subsequent cross-validated (leave one out) group prediction. The quality of the DA function is expressed by its canonical correlation (R*).

Principal component analyses (PCAs) were computed from MAP obtained from NOD mice with the purpose being to uncover the latent structure within protein families. Protein family membership was defined based on the Protein ANalysis THrough Evolutionary Relationships (PANTHER) classification system [24]. PCA seeks a linear combination of variables so that the maximum variance is extracted from the variables. It then removes this variance and seeks a second linear combination, and so forth. Loadings greater than 0.6 were considered defining parts of the component. For proper model specification, variables being either differentially expressed between the two strains (*P *< 0.05) and/or significantly correlated (r > 0.6; *P *< 0.05) with one of the disease parameters were included. As a rotation method, Varimax was chosen. The number of components was determined using the Kaiser criterion (Eigenvalue > 1.0). An explanatory criterion (>80%) was applied, in addition, for growth factors and cytokines in serum. Variables being defining parts of a component were also combined for DA and entered simultaneously. In serum no data were missing and in saliva missing values were excluded pair-wise from all analyses except PCA and DA. All analyses were computed using SPSS 13 (SPSS Inc., Chicago, IL, USA).

## Results

### Autoimmune disease manifestations

At 21 weeks of age salivary secretion (expressed as μl/minute per g bodyweight) in NOD mice (*n *= 12; 0.367 ± 0.026) was decreased by 42% compared with Balb/c mice (*n *= 12; 0.637 ± 0.024; *P *< 0.001; Additional file 1 [Supplementary table 2]). Salivary secretion rate classified 100% of the mice according to their strain membership, confirming the onset of overt SS in all NOD mice.

FS averaged 1.007 ± 0.087 foci/mm^2 ^and RI 0.045 ± 0.007 mm^2^/mm^2 ^in NOD, whereas Balb/c mice were free from glandular inflammation. IS in nondiabetic NOD mice averaged 0.454 ± 0.052 (Additional file 1 [Supplementary table 2]), representing mild to intermediate insulitis expressed on a scale from 0 (no inflammation) to 1 (all islets are to a large extent invaded by lymphocytes). Importantly, among all variables only serum glutamic-oxaloacetic transaminase (SGOT; r = -0.615, *P *= 0.033), serum IgA (r = -0.581, *P *= 0.048) and anti-M3R IgG_1 _(r = 0.702, *P *= 0.011) correlated with IS.

Histopathological evaluation of the kidneys, thyroid gland, thymus, heart, lungs, liver, stomach, small and large intestines, appendix and skin revealed a subset of NOD mice exhibiting mononuclear cell infiltration in the kidneys (*n *= 5; Additional file 1 [Supplementary figure 1A]), accompanied by hyaline casts in two cases (Additional file 1 [Supplementary figure 1B]). In one case, hyaline casts were found in the absence of lymphoid infiltration. In addition, some kidneys exhibited glomeruli with increased numbers of mesangial cells. In one kidney hyaline material was found in glomerular capillaries (Additional file 1 [Supplementary figure 1C]). Necrosis or crescent formation in the glomeruli, however, was not observed. NOD mice exhibiting signs of kidney pathology (*n *= 6) had significantly lower β_2_-microglobulin and lower anti-proteinase 3 antibody levels compared with mice free from such alterations (*n *= 6). Sections of lungs from 11 NOD mice presented foamy cells in the alveoli (Additioinal file 1 [Supplementary figure 1D, E]), which in three cases were accompanied by focal lymphoid infiltrates in the lungs (Additional file 1 [Supplementary figure 1D, F]). However, convincing histological patterns of interstitial lung disease were absent. Using light microscopic screening, no signs of other extraglandular disease manifestations or other independent autoimmune diseases were found.

### Univariate analyses

In serum the levels of 87 biomarkers, including 14 autoantibodies and four anti-M3R antibody subclasses, were measured (Additional file 1 [Supplementary table 2]). Ten proteins, mostly cytokines, were undetectable in serum of NOD and Balb/c. Interferon-γ was detectable in one NOD mouse and circulating fibroblast growth factor-9 in one Balb/c mouse only. In saliva 75 biomarkers were analyzed, of which IL-3 and leptin were undetectable in Balb/c mice; IL-3, leptin and glutathione S-transferase-α were detectable in only one, four and four NOD mice, respectively (Additional file 1 [Supplementary table 2]). Thirty-eight of the analytes assessed in serum were found at significantly different concentrations in NOD mice compared with Balb/c mice, whereas in saliva 34 analytes were significantly different (Table 1).

**Table 1:** Significantly different biomarker concentrations in NOD and Balb/c mice

Serum	Fold change	*t*-test *P *value	Saliva	Fold change	*t*-test *P *value
MIP-2 (CXCL-2)	0.74	0.0130	GCP-2 (CXCL-5)	1.81	< 0.0001
IP-10 (CXCL-10)	1.70	0.0145	MIP-2 (CXCL-2)	1.83	0.0236
LTN (XCL-1)	1.40	0.0037	IP-10 (CXCL-10)	4.58	0.0001
MCP-1 (CCL-2)	1.73	0.0007	LTN (XCL-1)	2.21	0.0204
MCP-3 (CCL-7)	1.99	0.0006	MCP-1 (CCL-2)	3.95	0.0304
MIP-1α (CCL-3)	1.37	0.0059	MCP-3 (CCL-7)	2.97	0.0024
MIP-1γ (CCL-9)	1.43	< 0.0001	MIP-1β (CCL-4)	3.14	0.0155
MDC (CCL-22)	1.28	0.0002	RANTES (CCL-5)	4.32	< 0.0001
MIP-3β (CCL-19)	1.57	0.0004	Eotaxin (CCL-11)	2.80	0.0179
OPN (SPP-1)	2.32	< 0.0001	MDC (CCL-22)	2.65	0.0102
IL-10	1.17	0.0201	IL-17	2.27	0.0294
CD40L	0.61	0.0136	GM-CSF (CSF-2)	2.06	0.0377
CD40	1.59	0.0007	IL-7	2.52	0.0244
IL-1α	0.67	0.0003	IL-10	1.36	0.0351
IL-18	1.58	0.0005	CD40	6.31	0.0277
EGF	1.44	0.0111	IL-11	12.18	0.0018
Growth hormone	3.41	0.0001	LIF	2.99	0.0116
SCF (Kitl)	1.29	0.0092	OSM	3.99	0.0102
VEGF-A	0.64	0.0051	IL-18	2.62	0.0193
Endothelin-1	1.64	0.0184	FGF-9 (ng/ml)	2.82	0.0021
Insulin	1.48	< 0.0001	M-CSF (CSF-1)	1.68	0.0289
CRP	1.85	< 0.0001	SCF (Kitl)	2.27	0.0349
Haptoglobin	1.14	0.0359	TPO	5.40	0.0003
SAP	1.43	< 0.0001	Leptin	-	0.0374
SGOT	1.33	0.0062	VCAM-1	1.49	0.0424
Factor III	1.45	0.0173	MMP-9	1.72	0.0191
Factor VII	1.54	0.0092	TIMP-1	1.58	0.0444
Fibrinogen	11.88	< 0.0001	MPO	1.97	0.0100
VCAM-1	1.39	< 0.0001	Anti-Insulin	1.32	0.0415
MMP-9	0.60	0.0170	SSB (anti-La)	1.20	0.0155
Cystatin-C	1.56	< 0.0001	Anti-RNP	1.46	0.0174
MPO	1.67	0.0001	Anti-beta 2GPI	1.56	0.0117
Apo A1	1.20	0.0024	Anti-mitochondrial	1.72	0.0392
IgA	0.47	< 0.0001	Anti-SCL70	1.40	0.0021
Anti-M3R IgG_1_	18.56	0.0029			
Anti-M3R IgG_2b_	11.23	0.0019			
Anti-M3R IgG_2c_	77.28	< 0.0001			
Anti-M3R IgG_3_	2.14	0.0219			

Fold change in biomarker concentrations between nonobese diabetic (NOD) and Balb/c mice. Proteins significantly different between the two strains (*P *< 0.05) are listed. For the remaining proteins, please refer to Additional file 1 (Supplementary table 2). Abbreviations not defined in the text: Apo, apolipoprotein; beta 2GPI, β_2_-glycoprotein; EGF, epidermal growth factor; FGF, fibroblast growth factor; GM-CSF, granulocyte macrophage colony-stimulating factor; Kitl, Kit ligand; LIF, leukaemia inhibitory factor; LTN, lymphotactin; M-CSF, macrophage colony-stimulating factor; MPO, myeloperoxidase; OPN, osteopontin; OSM, oncostatin M; SAP, serum amyloid P; SCF, stem cell factor; SCL, scleroderoderma; TPO, thrombopoietin; VEGF, vascular endothelial cell growth factor.

DA were subsequently computed to investigate each analyte's individual potential and relative importance in accurately predicting mouse strain. Cross-validated classification revealed 18 biomarkers in serum that predicted strain membership with 80% to 100% accuracy (hit rate) and with specificity and sensitivity up to 100%, whereas such capacity was identified for three chemokines measured in saliva (Table 2). Compared with nonvalidated group prediction, cross-validated prediction is based on all cases except the case being classified and it is thought to give a better estimate of the hit rate in the population. For each analyte shown in Table 2 the specificity (percentage of correct predictions in the NOD group) and sensitivity (percentage of correct predictions in the Balb/c group) were calculated.

**Table 2:** Strain membership-prediction potential of individual biomarkers

	R*	Specificity	Sensitivity	Hit rate
Salivary flow	0.849	100%	100%	100%
Serum				
IgA	0.921	100%	100%	100%
Anti-M3R IgG_2c_	0.902	92%	100%	96%
Cystatin-C	0.856	83%	92%	88%
Insulin	0.837	92%	83%	88%
CRP	0.812	92%	92%	92%
MIP-1γ (CCL-9)	0.784	100%	92%	96%
VCAM-1	0.783	92%	92%	92%
OPN (SSP-1)	0.778	83%	92%	88%
SAP	0.757	92%	83%	88%
Fibrinogen	0.747	83%	100%	92%
MPO	0.708	75%	92%	83%
Growth hormone	0.703	67%	100%	83%
MDC (CCL-22)	0.693	75%	92%	83%
IL-1α	0.678	83%	83%	83%
MIP-3β (CCL-19)	0.665	92%	92%	92%
IL-18	0.653	75%	100%	88%
MCP-3 (CCL-7)	0.648	67%	92%	79%
CD40	0.645	67%	92%	79%
MCP-1 (CCL-2)	0.643	67%	100%	83%
Anti-M3R IgG2b	0.600	67%	100%	83%
Saliva				
RANTES (CCL-5)	0.799	75%	92%	83%
GCP-2 (CXCL-5)	0.745	75%	92%	83%
IP-10 (CXCL-10)	0.731	75%	100%	88%
TPO	0.678	67%	83%	75%
Anti-SCL70	0.645	50%	100%	80%
IL-11	0.603	58%	92%	75%

Results from uni-variate DA sorted according to their canonical correlation. Only predictors yielding a canonical correlation >0.6 were included. Specificity (percentage of correct predictions in the NOD group), sensitivity (percentage of correct predictions in the Balb/c group) and hit ratio (% of correctly classified cases) represent results obtained from cross-validated (leave-one-out) group prediction analyses. CRP, C-reactive protein; MPO, myeloperoxidase; OPN, osteopontin; SAP, serum amyloid P; TPO, thrombopoietin.

### Multivariate analyses

**Figure 1:**
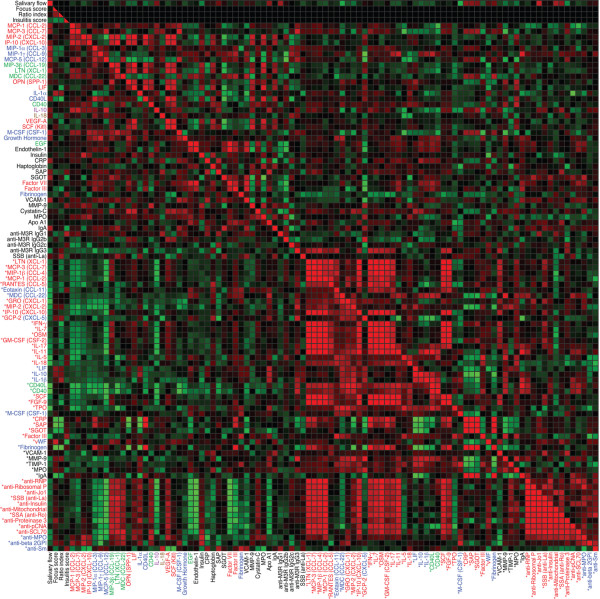
Correlation matrix from original variables measured in NOD and Balb/c mice. Correlation matrix of proteins differently expressed (*P *< 0.05) between nonobese diabetic (NOD) and Balb/c mice or significantly associated with autoimmune manifestations in NOD (r > 0.6, *P *< 0.05) sorted according to protein family membership and principal component structure. The lower left triangle displays the coefficients obtained from NOD, and the upper right triangle the values obtained from Balb/c mice. Red indicates positive correlation, and green negative correlation. Colour saturation indicates the strength of the association. Protein names printed in black are variables that could not be fitted in principal component analyses. Red lettering identifies the variable as being a significant part of component 1, blue of component 2, green of component 3, and so on, of the corresponding of protein family. *Variables representing measurements in saliva. The figure was drawn using iVici 0.91. Abbreviations not defined in the text: Apo, apolipoprotein; beta 2GPI, β_2_-glycoprotein; CRP, C-reactive protein; EGF, epidermal growth factor; FGF, fibroblast growth factor; GM-CSF, granulocyte macrophage colony-stimulating factor; GRO, melanoma growth stimulatory activity protein; LIF, leukaemia inhibitory factor; LTN, lymphotactin; M-CSF, macrophage-colony stimulating factor; MPO, myeloperoxidase; OPN, osteopontin; OSM, oncostatin M; SAP, serum amyloid P; SCF, stem cell factor; SCL, scleroderoderma; TIMP, tissue inhibitor of metalloproteinase; TPO, thrombopoietin; VEGF, vascular endothelial cell growth factor.

An immune response is an orchestrated process that involves several protein families and several molecules with similar molecular function. The PANTHER classification system was used to classify the analytes into families of proteins with shared function based on scientific experimental evidence and evolutionary relationships. A correlation matrix comprising the variables identified to be suitable for modelling purposes exhibited profound differences in protein associations within and among protein families (Figure 1). To gain further understanding of these interrelationships in NOD mice, PCA was computed to uncover the underlying dimensions of the immune response (Additional file 1 [Supplementary table 3]). PCA identifies patterns in data and uses a correlation matrix to find the linear combination of original variables, which accounts for most of the variance. It then represents those objects in terms of these new linear combinations, which are called principal components. The first component will be defined to account for the maximum of variation possible (Additional file 1 [Supplementary figure 3]). The second component will subsequently account for the maximum of the remaining variation, and so forth, until a certain criterion set by the researcher is met. In our dataset such patterns were clearly recognizable (Figure 1). Consequently, a large number of the original variables, entered according to protein family membership, could be combined into 27 components that still accounted for at least 80% of the original variance (Additional file 1 [Supplementary table 3]).

**Figure 2:**
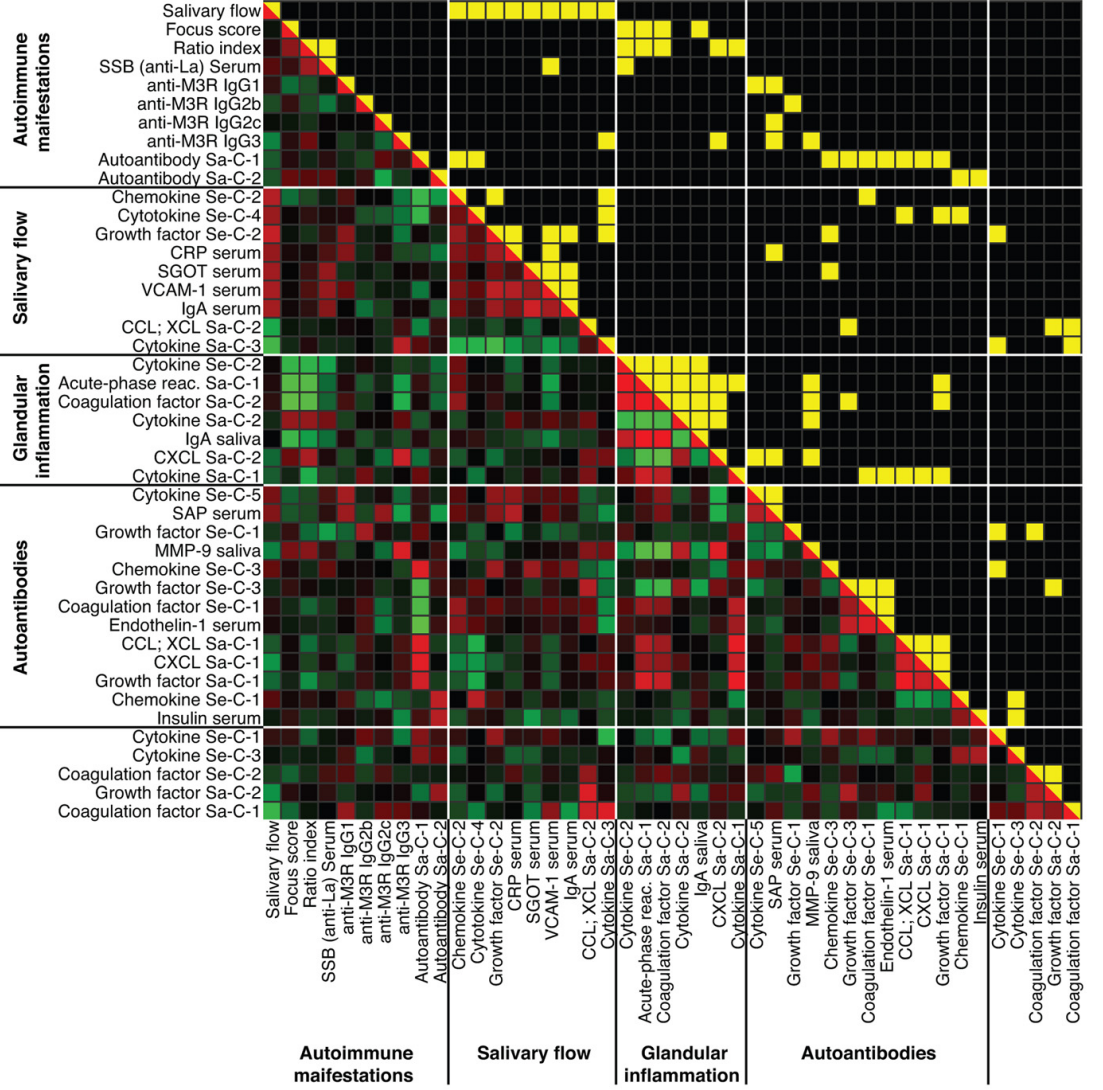
Correlation matrix of principal components and original variables associated with autoimmune manifestations. Correlation matrix of principal components and original variables sorted according to their associations with Sjögren's syndrome (SS) disease manifestations. The upper right triangle indicates significant *P *values with yellow fill (*P *< 0.05). The lower left triangle displays the corresponding r. Red indicates positive correlation and green negative correlation, and colour saturation indicates the strength of the association. Abbreviations not defined in the text: CRP, C-reactive protein; SAP, serum amyloid P.

**Figure 3:**
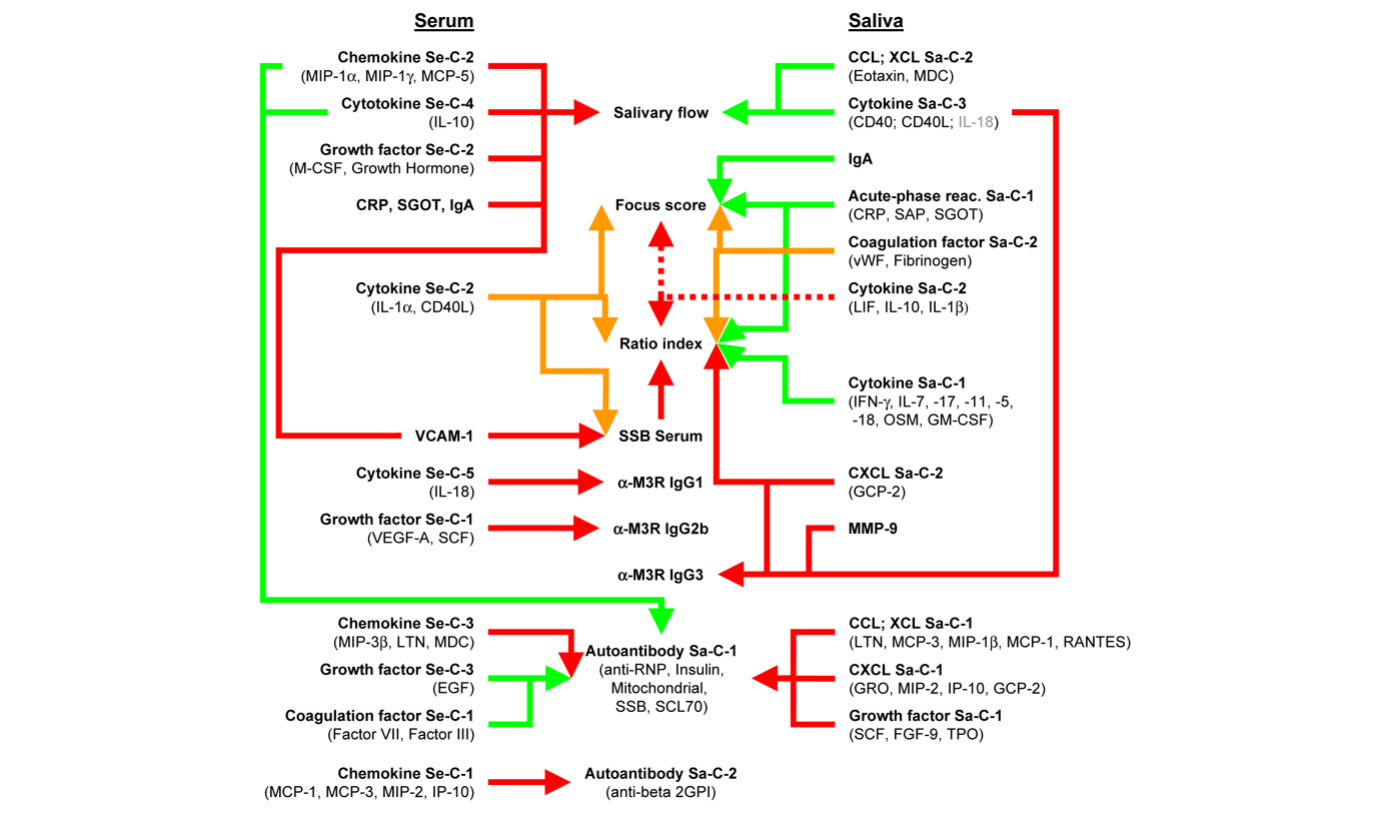
Schematic map of principal component associations. Model of principal component associations and selected original variables with autoimmune manifestations. Red arrows mark significant positive correlations and green arrows significant negative correlations. Orange arrows represent significant associations of components with significant positive and negative loadings. The defining variables of the components are given in parentheses. Dotted lines and grey lettering mark borderline significance. The associations involving circulating serum amyloid P (SAP), endothelin-1 and insulin are not shown in the figure; please refer to Figure 2. Abbreviations not defined in the text: beta 2GPI, β_2_-glycoprotein; CRP, C-reactive protein; EGF, epidermal growth factor; FGF, fibroblast growth factor; GM-CSF, granulocyte macrophage colony-stimulating factor; GRO, melanoma growth stimulatory activity protein; IFN, interferon; LIF, leukaemia inhibitory factor; LTN, lymphotactin; M-CSF, macrophage-colony stimulating factor; OSM, oncostatin M; SCF, stem cell factor; SCL, scleroderoderma; TPO, thrombopoietin; VEGF, vascular endothelial cell growth factor.

**Figure 4:**
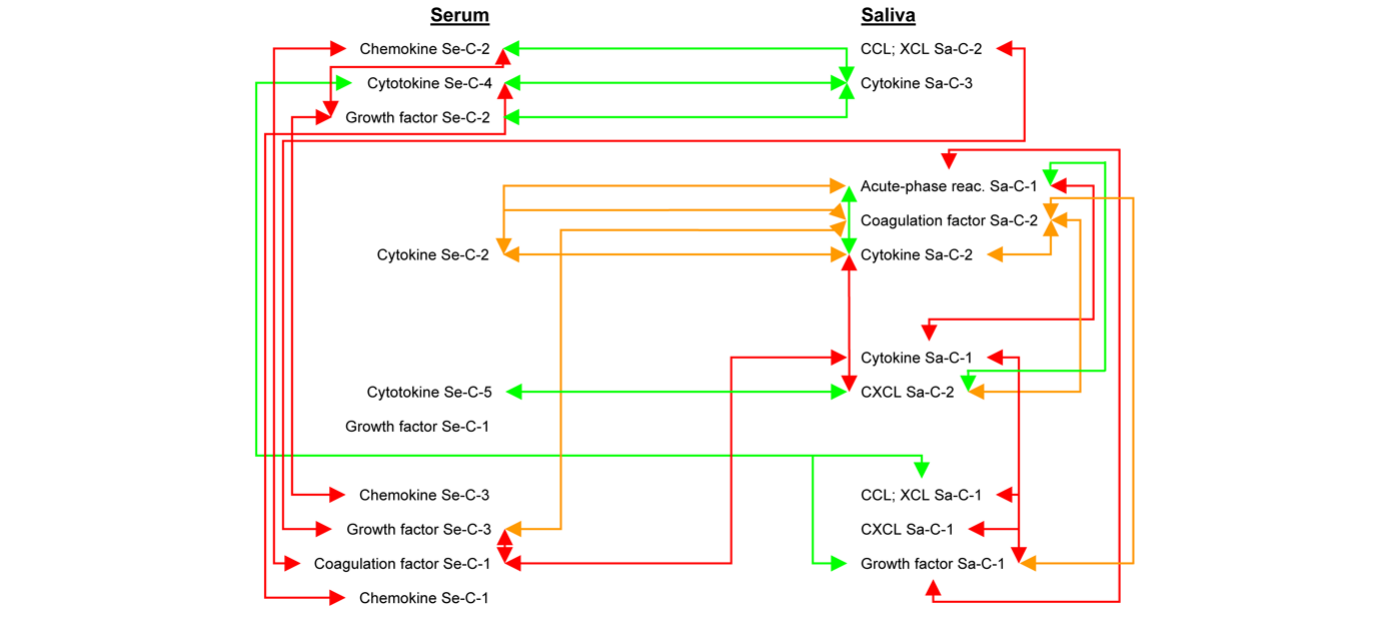
Schematic map of principal component associations. Model of bidirectional inter-component associations. Red arrows mark significant positive correlations and green arrows significant negative correlations. Orange arrows represent interrelations of components of which at least one had significant positive and negative loadings.

Subsequently, linear interrelationships were analyzed using Pearson correlation. The extraction of principal components reduced the correlation matrix considerably, from 9'604 (Figure 1) to 1'994 coefficients (Figure 2). Components associated with disease manifestations are presented in Figures 2 and 3, whereas correlations between components are shown in Figures 2 and 4. Variables for which no reasonable component structure could be computed were included as original variables, if a significant correlation with an autoimmune manifestation was detected (Figures 2 and 3). Results form multivariate DA, based on the simultaneous entry of the variables combined in the different components, are listed in Additional file 1 (Supplementary table 4). They represent the relative importance of the collinear variables, combined in an individual component, in predicting strain membership.

### Salivary flow

Correlation analyses indicated no linear association between salivary flow and the parameters of glandular inflammation (Figure 2 and 3). PCA identified three components in serum (Se-C) and two in saliva (Sa-C) that correlated with salivary flow (Figure 3). The defining variables of the components are specified in parentheses. Chemokine Se-C-2 (macrophage-inflammatory protein [MIP]-1α, MIP-1γ and monocyte chemoattractant protein [MCP]-5), cytokine Se-C-4 (IL-10) and growth factor Se-C-2 (macrophage-colony stimulating factor and growth hormone) were all positively correlated with salivary flow (Figure 3 and Additional file 1 [Supplementary table 3]). The same applied for the following serum analytes, for which no PCA-based solution could be computed: C-reactive protein, SGOT, vascular cell adhesion molecule (VCAM)-1 and IgA.

In saliva, C-C chemokine ligand/C chemokine ligand Sa-C-2 (eotaxin and macrophage-derived chemokine [MDC]) and cytokine Sa-C-3 (CD40 and CD40L [IL-18 borderline]) correlated with decreased salivary flow. Not reflected by their corresponding component, salivary IL-5 (r = -0.708, *P *= 0.010), thrombopoietin (r = -0.766, *P *= 0.004) and factor III (r = -0.614, *P *= 0.034) also correlated with salivary flow.

### Glandular inflammation

Glandular inflammation was negatively correlated with cytokine Se-C-2 (negative loading for IL-1α [-0.94] and positive loading for CD40 ligand [CD40L; 0.68]). Interestingly, this component was correlated with SSB in serum.

In saliva multiple factors exhibited a linear interrelationship with measures of glandular inflammation. Acute phase reactants Sa-C-1 (C-reactive protein, SGOT and serum amyloid P) and coagulation factor Sa-C-2 (von Willebrand factor [vWF] and fibrinogen) correlated negatively with FS and RI. vWF was negatively loaded on coagulation factor Sa-C-2, consistent with its initial positive correlation with increased IR (r = 0.792, *P *= 0.002). In addition, secretory IgA was inversely correlated with FS. Components extracted from protein families involved in specific immune reactions revealed C-X-C chemokine ligand (CXCL) Sa-C-2 (granulocyte chemotactic protein [GCP]-2) to be positively correlated with IR (r = 0.664, *P *= 0.018), and cytokine Sa-C-1, combining eight cytokines, exhibited a negative association with IR. In contrast, cytokine Sa-C-2 (leukaemia inhibitory factor, IL-10 and IL-1β) showed an almost significant positive correlation with FS and IR (both r = 0.581, *P *= 0.061). Correlation patterns compared with other components further supports its connection with glandular inflammation (Figure 2).

### Autoantibodies

We found all isotypes of M3R autoantibodies that we investigated to be significantly increased in NOD mice compared with Balb/c mice. Circulating SSB was the only autoantibody that significantly correlated with any SS disease manifestation. PCA failed to extract components from circulating autoantibody levels, and therefore individual associations are reported in Figures 2 and 3. Interestingly, SSB levels were associated with cytokine Se-C-2 (r = -0.590, *P *= 0.043) and VCAM-1 (r = 0.587, *P *= 0.045) and anti-M3R IgG_1 _correlated with cytokine Se-C-5 (IL-18; r = 0.649, *P *= 0.023). Levels of anti-M3R IgG_3 _correlated negatively with MIP-1α (r = -0.626, *P *= 0.029) and MCP-5 (r = -0.580, *P *= 0.048), which in turn were associated with salivary flow (r = 0.735, *P *= 0.006 and r = 0.742, *P *= 0.006, respectively). Consistent with this observation, levels of anti-M3R IgG_3 _correlated with cytokine Sa-C-3 (CD40, CD40L and borderline IL-18; r = 0.724, *P *= 0.012) on its part associated with decreased salivary flow. In addition, the positive association between M3R IgG_3 _and CXCL Sa-C-2 (GCP-2; r = 0.789, *P *= 0.002) in its turn correlating with RI, suggests that anti-M3R IgG_3 _has a role in connecting different SS-related disease manifestations. In addition, salivary matrix metalloproteinase (MMP)-9 also correlated positively with increased anti-M3R IgG3.

Autoantibodies in saliva were grouped into autoantibody Sa-C-1 (anti-RNP, anti-insulin, anti-mitochondrial, SSB and anti-scleroderma-70 antibodies) and autoantibody Sa-C-2 (anti-β_2 _glycoprotein). Components positively correlated with autoantibody Sa-C-1 were chemokine Se-C-3 (MIP-3β, lymphotactin and MDC), C-C chemokine ligand/XCL Sa-C-1 (lymphotactin, MCP-3, MIP-1β, MCP-1 and RANTES [regulated upon activation, normal T-cell expressed and secreted]), CXCL Sa-C-1 (melanoma growth stimulatory-activity protein, MIP-2, inducible protein [IP]-10 and GCP-2) and growth factor Sa-C-1 (stem-cell factor, fibroblast growth factor-9 and thrombopoietin). In contrast, chemokine Se-C-2 and cytokine Se-C-4, in turn also associated with salivary flow, exhibited a significant negative correlation with autoantibody Sa-C-1. Growth factor Se-C-3 (epidermal growth factor) and coagulation factor Se-C-1 (factors VII and III), together with endothelin-1, correlated negatively with autoantibody Sa-C-1 as well. Autoantibody Sa-C-2 correlated positively with chemokine Se-C-1 (MCP-1, MCP-3, MIP-2 and IP-10) and circulating insulin.

In summary, proteins were mostly associated exclusively with either salivary flow, parameters of glandular inflammation, or autoantibody levels. Dual associations were only apparent for the following: chemokine Se-C-2, cytokine Se-C-4, cytokine Se-C-2, cytokine Sa-C-3 and CXCL Sa-C-2. From variables not included in PCA, only VCAM-1 in serum correlated with two autoimmune parameters.

### Associations among components

Correlations among components associated with autoimmune manifestations are presented in Figures 2 and 4. Between components associated with salivary flow, an antagonistic interplay between cytokine Sa-C-3 (associated with decreased salivary flow) and all three components extracted from serum (positively associated with salivary flow) was apparent. Components associated with FS and/or RI correlated strongly with each other, indicating a process in which cytokine Sa-C-2 and CXCL Sa-C-2 oppose acute-phase reactant Sa-C-1 and secreted IgA. In addition, cytokine Se-C-2 and coagulation factor Sa-C-2 played a dual role by combining the positively associated IL-1α and vWF with the negatively associated CD40L and fibrinogen, respectively. Regarding components associated with autoantibodies, the components related to anti-M3R IgG_1 _correlated negatively with autoantibody Sa-C-1, whereas all components that were positively associated with autoantibody Sa-C-1 correlated with each other.

We observed a complete absence of original variables or components that correlated with salivary flow and either FS or RI. This absence was as absolute when analyzing inter-component correlations (Figure 2). In contrast, some components were associated with either salivary flow or glandular inflammation, having positive or negative inter-relationships with components related to autoantibody levels, such as cytokine Se-C-4 (IL-10) and cytokine Sa-C-1 (interferon-γ, IL-7, oncostatin M, granulocyte macrophage-colony stimulating factor, IL-17, IL-11, IL-5 and IL-18). CXCL Sa-C-2 (GCP-2) exhibited negative associations with the proteins related to increased anti-M3R IgG_1 _and positive associations with anti-M3R IgG_3 _and MMP-9. Interestingly, MMP-9 expression correlated positively with components associated with increased glandular inflammation.

## Discussion

By studying the immune system through the application of reductionist principles, its mediators have been thoroughly analyzed over recent decades, which has yielded tremendous scientific advances. However, studying the properties of its isolated components is limited in terms of elucidating how system properties emerge, because they may strongly rely on and arise from interactions between its numerous constituents. In this study we present a substantial amount of data on immunologically relevant proteins, for which often only scant or no information within the context with SS has been published. Multiplexing of analytes thus significantly diminished the sample volume required and enabled us to investigate the analytes' connectivity through correlation networks. Such a study cannot be as conclusive in defining the role of a single protein as a component-focused experimental study design. However, it represents a novel way to analyze the implications of multiple molecules in a specific condition, by providing insight into the inter-relationships that define a specific system state.

Analyses of protein levels instead of mRNA expression data can allow exclusion of certain factors that cause uncertainty, such as RNA stability and correlations between mRNA levels and corresponding protein levels. A recent study combined global gene expression analyses with quantitative proteomics based on two-dimensional gel electrophoresis and mass spectrometry in saliva obtained from patients with SS [25]. Indeed, the correlation between mRNA levels and proteins was proven to be poor. Using mass spectrometry, 42 proteins were identified that were significantly altered when comparing pooled saliva from SS patients with healthy control individuals [25]. None of the proteins identified by Hu and coworkers [25] was included in our MAP. Two-dimensional gel electrophoresis is limited in terms of its sensitivity in identifying proteins with concentrations in the pg/ml range, especially if they may be masked by abundant proteins present in the biofluid of interest. However, methods such as stable-isotope protein tagging or subtractive proteomics may improve the number of immune system related proteins identified by mass spectrometry. Nevertheless, the two approaches of antibody-based biomarker identification and mass spectrometry-based global proteome profiling may well complement one another in delineating SS-specific disease signatures. Nevertheless, further technological advances are required in both fields to achieve more complete coverage of crucial immune mediators. Unfortunately, molecules of proven importance in SS, such as B-cell activating factor [26] and type I interferons [27] were not part of our MAP. Including such crucial molecules in future studies and the present correlation network would indeed greatly increase completeness and enahce the plausibility of an integrated model of SS.

By accounting for SS-related extraglandular autoimmune manifestations and insulitis, we are confident that analyses in serum reflect to a large extent systemic aspects of SS. Nevertheless, although we could not associate the presence of extraglandular disease manifestations with the situation in the salivary glands, it would be of interest to investigate further the involvement of kidneys and lungs in both experimental and human SS [28-30]. The potential presence of SS-unrelated histopathology or of other subclinical autoimmune diseases in mouse models for SS represents a common problem of all mouse models used in SS research [13,31]. This divergence of these models from clinical SS may lead one to draw erroneous conclusions from measurements in serum. Indeed, biomarker profiling solely focusing on serum or plasma may require considerable validation efforts to prove their specificity for a specific autoimmune disease such as SS.

In contrast, because it is directly collected from the site of inflammation, saliva may largely reflect the immunological situation in the salivary glands. The presence and local production of some inflammatory mediators and autoantibodies in the salivary glands were previously described (Additional file 1 [Supplementary table 1]); these findings were largely confirmed by our study. Importantly, we did not observe a concentration effect related to lesser fluid secretion. In a noninflammatory situation (represented by Balb/c mice), the average correlation coefficient of analytes included in the model did not show a negative association with salivary flow (r = -0.076). The noninvasive collection method and the lack of extraction procedures predispose biomarkers, measured in saliva, as potential surrogate markers of disease and disease activity [25,32]. In addition, they may reflect the disease independent from other inflammatory conditions in patients. The identification of a set of biomarkers in human saliva, similar to the three chemokines we identified in mice, that predicts the presence of glandular inflammation with high accuracy would represent a major advance in the field.

Traditionally, loss of secretory capacity, degree of lymphoid infiltration and production of specific autoantibodies have been anticipated to correlate with each other and to indicate disease state and severity [5,6]. However, the correctness of this assumption was difficult to prove. Our findings strongly argue against such close interrelationships and suggest that there is much independence of the various hallmarks of SS. Only RI and SSB correlated directly with each other, and the separation between proteins in terms of whether they were associated with hyposalivation or with glandular inflammation was absolute.

Circulating MIP-1α and MCP-5, which we found to be associated with higher salivary flow, are Th1-related chemokines that are negatively regulated by STAT6 [33]. In opposition, eotaxin and MDC, correlating with decreased salivary flow, are dependent on STAT6 [33] and are considered to be Th2-related chemokines [34]. In accordance with these findings, STAT6-deficient NOD mice did not develop hyposalivation [20]. Both eotaxin and MDC are produced by Th2-promoting dendritic cell types upon engagement of CD40/CD40L [35]; in our study, these two proteins were associated with low salivary secretion capacity as well. CD40 and CD40L are expressed on salivary gland epithelial cells and infiltrating lymphocytes in biopsies obtained from SS patients [36]. The observed correlation between CD40/CD40L and anti-M3R IgG_3 _levels may therefore be related to the primary role played by CD40 and CD40L in B-cell survival, B-cell proliferation, antibody production and antibody isotype switching [37]. We found all of the antibody isotypes binding M3R that we measured to be upregulated, with (perhaps most importantly) positive associations between M3R IgG_3 _and secretory CD40, CD40L, IL-18, GCP-2 and MMP-9. Previous studies identified IgG_1 _as the crucial isotype in anti-M3R antibody mediated inhibition of salivary flow in STAT6^-/- ^[20] and IL4^-/- ^[23] deficient NOD mice. Nevertheless, IgG_2 _subclasses and IgG_3 _are generally considered to be significantly more potent in mediating pathogenic effects [38]. In contrast to other isotypes, the effect of IgG_3 _is FcR independent and strictly related to complement activation; the latter component of the immune system was recently related to SS pathology [39]. In addition, IgG_3 _can form complexes through self-association and generate cryoglobulins [38], a feature observed in SS [6]. With regard to the quality of the antibody response, we found total circulating and secretory IgA to be related to lower disease activity.

Apart from B-cell fate, CD40/CD40L ligation plays a central role in converting a tolerogenic antigen presentation into pathogenic immune activation [40]. In conditions with chronic inflammation, CD40 and other inflammatory mediators can have adverse effects on tissue renewal and repair processes [41]. Indeed, we found specific growth factors to be negatively correlated with CD40 and CD40L and secreted autoantibodies. Furthermore, we found circulating IL-10 to correlate negatively with CD40/CD40L; the anti-inflammatory properties of IL-10 may apply to our model, because increased circulating IL-10 did correlate with higher salivary flow and lower autoantibody concentrations in saliva. IL-10 gene transfer in NOD mice partially suppressed the appearance of SS-like features [42]. However, we found IL-10 in saliva to be associated with glandular inflammation, which corroborates reports indicating that IL-10 transgenic mice develop progressive histopathology and hyposalivation, evocative of SS [43].

With the exception of vWF, increased concentrations of proteins in saliva related to acute tissue injury were all associated with a lower degree of glandular inflammation. These events may be followed later by inflammatory cell invasion into the glandular tissue and/or mirror an imbalance between processes that restore homeostasis and factors that promote chronic inflammation. Higher levels of IL-1 family members did correlate with both worsening of hyposalivation and increased glandular inflammation. IL-1β is also a major inducer of GCP-2 [44] and was, similar to IL-1β, associated with glandular inflammation. Leukaemia inhibitory factor, inducible through IL-1β [45] and loaded on the same component together with IL-1β and IL-10, has been shown to have parallels with IL-1, tumour necrosis factor-α and IL-6 within the context of promoting inflammation in rheumatoid arthritis [45].

Among the biomarkers analyzed in saliva, GCP-2, IP-10 and RANTES exhibited the greatest potential in predicting strain membership. GCP-2 is expressed at neutrophil and macrophage dominated inflammatory sites, IP-10 is related to Th1 immune responses, and RANTES can be involved in Th1 and Th2 immune responses [46]. GCP-2 and IP-10 have opposite roles in angiogenesis [47]; this issue has not yet been addressed in SS, despite the recognized importance of neovascularization in promoting the influx of inflammatory cells [48]. Proteins such as GCP-2, vascular endothelial growth factor, epidermal growth factor, IL-1, VCAM-1 and MMP-9, which we found to be significantly increased in NOD mice, are involved in angiogenesis [48]. In addition, angiostatic molecules such as IP-10, tissue inhibitor of metalloproteinase-1 and endostatin-1 were also altered [48]. Based on our findings, pathogenic neovascularization deserves to receive more attention in SS research.

## Conclusion

Increased concentrations of molecules that govern adaptive immune responses were associated with an aggravation of SS coupled with a strong association of Th2-related proteins with hyposalivation. In addition, we observed great independence among the different disease manifestations of SS, which must be considered when one is evaluating potential drug targets. Nevertheless, CD40, CD40L, IL-18, GCP-2 and anti-M3R IgG_3 _may, however, represent a pivotal point that intersects the various aspects of SS pathology. As multiplex technology allowed us to draw a comprehensive picture of the overt disease stage, we suggest that treatment outcome may similarly be monitored [49]. Hypothesis-driven verification of our findings, together with validation of our findings in human SS specimens, are important goals for the future. We also believe that discovery-driven studies, such as the one presented here, can widen the horizon and inspire new component-focused basic research. Nevertheless, bridging the gap between these two approaches represents a major challenge for the years to come, but the reward will be a more integrated perspective on immunology and autoimmune disease.

## Abbreviations

CD40L = CD40 ligand; CXCL = C-X-C chemokine ligand; DA = discriminant analyses; FITC = fluorescein isothiocyanate; FS = focus score; GCP = granulocyte chemotactic protein; IL = interleukin; IP = inducible protein; IS = insulitis score; MAP = multi-analyte profile; MCP = monocyte chemoattractant protein; MDC = macrophage-derived chemokine; MIP = macrophage-inflammatory protein; MMP = matrix metalloproteinase; M3R = M3 receptor; NOD = nonobese diabetic; PANTHER = Protein ANalysis THrough Evolutionary Relationships; PCA = principal component analysis; RANTES = regulated upon activation, normal T-cell expressed and secreted; RI = ratio index; SGOT = serum glutamic-oxaloacetic transaminase; SS = Sjögren's syndrome; STAT = signal transducer and activator of transcription; Th = T-helper; VCAM = vascular cell adhesion molecule; vWF = von Willebrand factor.

## Competing interests

The authors declare that they have no competing interests.

## Authors' contributions

ND and RJ designed the study. ND carried out the animal experiments, salivary flow measurements and collection of organs. HI participated in preparation and carried out the qualitative evaluation of the organs. ND carried out the morphometrical analyses. JC and ND carried out the fluorescence-activated cell sorting analyses for anti-M3R autoanatibodies. ND carried out the data analyses. ND and RJ wrote the manuscript. RJ supervised the study. All authors read and approved the final manuscript.

## Supplementary Material

Additional file 1 Supplementary table 1 provides a literature overview; Supplementary table 2 provides a comparison of disease parameters and analyte concentrations in serum and saliva; Supplementary table 3 provides principal component analyses (component structure and loadings); Supplementary table 4 shows strain membership prediction by defining variables from principal components; and Supplementary figure 1 shows extraglandular disease manifestations in the kidneys and the lungs present in a subset of NOD mice. Click here for file
