# Supplementary material for: Biomarker profiles in serum and saliva of experimental Sjögren's syndrome: associations with specific autoimmune manifestations
Source: Arthritis Res Ther. 2008 Feb 20;10(1):R22. doi: 10.1186/ar2375 (PMC2374466; doi:10.1186/ar2375)
Supplement: Additional file 1 — Supplementary table 1 provides a literature overview; Supplementary table 2 provides a comparison of disease parameters and analyte concentrations in serum and saliva; Supplementary table 3 provides principal component analyses (component structure and loadings); Supplementary table 4 shows strain membership prediction by defining variables from principal components; and Supplementary figure 1 shows extraglandular disease manifestations in the kidneys and the lungs present in a subset of NOD mice. [file ar2375-S1.pdf]

Supplementary table 1) Literature overview

	Human material			NOD and SS		
	**Serum**	**Saliva**	**Other**	**Serum**	**Saliva**	**Other**
IP-10 (CXCL-10)						[1]
LTN (XCL-1)						[1]
MCP-1 (CCL-2)	[2, 3]					
MIP-1α (CCL-3)	[2, 3]		[4]			
MIP-1β (CCL-4)	[2, 3]		[4]			
RANTES (CCL-5)	[2, 3]		[4]			[1, 5]
Eotaxin (CCL-11)	[2, 3]					
IFN-γ	[2, 3]	[6, 7]	[7-10]	[11]	[11]	[5, 12, 13]
IL-12p70	[2, 3]		[8, 10]	[11]	[11]	[5, 13]
IL-4	[2, 3]	[7]	[7, 8, 10]	[11, 13]	[11]	[5, 14]
IL-5	[2, 3]	[7]	[7]	[11]	[11]	
IL-17	[2, 3]					
GM-CSF (CSF-2)	[2, 3]		[9]	[11]	[11]	
IL-2	[2, 3]	[7]	[7-10]	[11, 13]	[11]	[5]
IL-10	[2, 3]	[7]	[7, 8]	[11, 13]	[11]	[5, 15]
TNF-α	[2, 3]	[7]	[7, 8]	[11]	[11]	[5]
CD40L	[16]	[16]	[17]			
CD40			[17]			
IL-1α	[2]	[7]	[7]			[13]
IL-1β			[8, 9]	[11]	[11]	
IL-18	[18, 19]		[10, 19]			
IL-6	[2, 3]	[6, 7]	[7-9]	[11, 13]	[11]	[5]
EGF	[3]		[20]		[21]	[12]
FGF-b (FGF-2)	[22]	[22]				
Growth Hormone	[23]		[23]			
VEGF-A	[3]					
CRP	[24, 25]					
Haptoglobin	[26]					
SGOT		[27]				
Fibrinogen	[25]		[28]			
vWF	[29]					
VCAM-1	[30]	[30]	[31]			[32]
MMP-9	[33, 34]	[35]	[33, 36]		[37]	[37, 38]
TIMP-1	[34]	[35]	[36]			
Cystatin-C		[39]				
MPO	[40]					
NGAL	[40]		[41]			
Apo-A1	[42, 43]					
B_2m	[44]	[39]	[45]			
Clusterin		[30]	[30]			
GST-Mu			[46]			
IgA	[47]	[48]	[49]		[50]	
anti-M3R	[51, 52]		[53]	[13]		
SSA (anti-Ro)	[54]	[55]	[56]	[57]		
SSB (anti-La)	[54]	[55]	[56]	[57]		
anti-RNP	[54, 58]					
anti-pCNA	[59]					
anti-Proteinase 3	[60]					
anti-Sm	[54, 58]					
anti-beta 2GPI	[61]					
anti-MPO	[60, 62]	[62]				
anti-Jo1	[58]					
anti-Mitochondrial	[63]					
anti-SCL70	[54, 58]					
anti-Ribosomal P	[64]					

Molecules included in this study analyzed previously in either, specimens obtained from patients with SS or reported in relation with SS-like pathology in NOD mice. The column “other” includes IHC on tissue sections, *in-situ* hybridization, RNA extracts etc. The search was limited to publications written in English and indexed in PubMed. The most common synonyms and MeSH terms were included in the search, which was finalized in June 2007. Abbreviations not introduced in the main manuscript: B2m (beta-2 microglobulin), GST (glutathione S-transferase), Jo-1 (histidyl-RNA synthetase), NGAL (lipocalin-2), pCNA (proliferating cell nuclear antigen), Sm (Smith), RNP (ribonuclear protein), TNF (tumor necrosis factor).

Supplementary table 2) Comparison of disease parameters and analyte concentrations in serum and saliva

	Strain					*t*-test	Strain					*t*-test
	Balb/c		NOD			(sig. 2-	Balb/c		NOD			(sig. 2-
**Pathology**	**Mean**	**SEM**	**Mean**	**SEM**	**Change**	**tailed)**	**Mean**	**SEM**	**Mean**	**SEM**	**Change**	**tailed)**
Salivary flow	0.64	(0.02)	0.37	(0.03)	-42%	< 0.0001						
Focus score	not present		1.01	(0.09)	-	-						
Ratio index	not present		0.05	(0.01)	-	-						
Insulitis score	not present		0.45	(0.05)	-	-						
			**Serum**						**Saliva**			
**Chemokines**												
GRO (CXCL-1) [ng/ml]	n/d		n/d		-	-	0.03	(0.01)	0.08	(0.03)	+147%	0.0825
GCP-2 (CXCL-5) [ng/ml]	18.8	(0.79)	18.0	(0.82)	-4%	0.4701	0.27	(0.03)	0.49	(0.03)	+81%	< 0.0001
MIP-2 (CXCL-2) [pg/ml]	25.8	(2.30)	19.1	(0.96)	-26%	0.0130	6.44	(1.07)	11.8	(1.92)	+83%	0.0236
IP-10 (CXCL-10) [pg/ml]	41.8	(4.05)	71.3	(10.3)	+70%	0.0145	8.81	(1.93)	40.3	(5.98)	+358%	0.0001
LTN (XCL-1) [pg/ml]	80.4	(6.77)	113	(7.22)	+40%	0.0037	11.3	(2.85)	25.0	(4.66)	+121%	0.0204
MCP-1 (CCL-2) [pg/ml]	72.3	(3.49)	125	(13.0)	+73%	0.0007	1.07	(0.58)	4.22	(1.23)	+295%	0.0304
MCP-3 (CCL-7) [pg/ml]	145	(11.9)	289	(34.0)	+99%	0.0006	4.08	(1.12)	12.1	(2.06)	+197%	0.0024
MCP-5 (CCL-12) [pg/ml]	42.9	(4.97)	45.2	(8.77)	+5%	0.8255	4.70	(4.40)	3.38	(1.01)	-28%	0.7733
MIP-1α (CCL-3) [pg/ml]	125	(13.8)	171	(5.96)	+37%	0.0059	29.0	(7.28)	37.1	(7.95)	+28%	0.4615
MIP-1β (CCL-4) [pg/ml]	66.2	(17.4)	118	(53.2)	+79%	0.3622	17.8	(6.21)	55.8	(13.1)	+214%	0.0155
MIP-1γ (CCL-9) [ng/ml]	9.73	(0.50)	13.9	(0.50)	+43%	< 0.0001	0.54	(0.11)	0.45	(0.05)	-17%	0.4468
RANTES (CCL-5) [pg/ml]	n/d		n/d		-	-	3.28	(0.85)	14.1	(1.52)	+332%	< 0.0001
Eotaxin (CCL-11) [pg/ml]	878	(67.2)	947	(58.5)	+8%	0.4420	1.61	(0.41)	4.50	(1.05)	+180%	0.0179
MDC (CCL-22) [pg/ml]	398	(16.6)	508	(17.7)	+28%	0.0002	7.62	(2.44)	20.2	(3.74)	+165%	0.0102
MIP-3β (CCL-19) [pg/ml]	409	(36.6)	643	(42.3)	+57%	0.0004	8.60	(5.02)	24.8	(7.13)	+188%	0.0771
**Cytokines (type-1)**												
IFN-γ [pg/ml]	n/d		0.63	(0.63)	-	0.3282	7.90	(2.36)	15.2	(3.22)	+92%	0.0812
IL-12p70 [pg/ml]	n/d		n/d		-	-	39.3	(15.1)	90.3	(23.6)	+130%	0.0825
OPN (SPP-1) [ng/ml]	91.5	(7.29)	212	(19.5)	+132%	< 0.0001	n/m		n/m		-	-
**Cytokines (type-2)**												
IL-4 [pg/ml]	n/d		n/d		-	-	30.3	(6.22)	44.7	(6.94)	+47%	0.1383
IL-5 [pg/ml]	33.0	(25.1)	37.5	(19.1)	+14%	0.1674	21.3	(6.25)	43.8	(13.7)	+106%	0.1485
**Cytokines (type-3)**												
IL-17 [pg/ml]	n/d		n/d		-	-	8.30	(2.61)	18.8	(3.71)	+127%	0.0294
**Pleiotropic cytokines**												
GM-CSF (CSF-2) [pg/ml]	n/d		n/d		-	-	0.90	(0.25)	1.85	(0.35)	+106%	0.0377
IL-2 [pg/ml]	n/d		n/d		-	-	10.2	(1.81)	14.6	(2.85)	+43%	0.2056
IL-3 [pg/ml]	n/d		n/d		-	-	n/d		0.18	(0.18)	-	0.3282
IL-7 [pg/ml]	17.0	(11.6)	7.10	(6.31)	-58%	0.4606	18.8	(6.30)	47.3	(9.94)	+152%	0.0244
IL-10 [pg/ml]	379	(20.5)	442	(14.2)	+17%	0.0201	153	(19.6)	209	(15.1)	+36%	0.0351
**TNF superfamily**												
TNF-α [pg/ml]	29.6	(8.27)	12.3	(3.57)	-58%	0.0677	n/d		1.70	(1.21)	-	0.1666
CD40L [pg/ml]	902	(102)	551	(82.6)	-39%	0.0136	24.3	(19.0)	114	(45.7)	+369%	0.0759
CD40 [pg/ml]	158	(8.76)	251	(21.9)	+59%	0.0007	1.29	(0.71)	8.15	(2.93)	+531%	0.0277
**IL-6 like cytokines**												
IL-6 [pg/ml]	n/d		n/d		-	-	1.10	(0.49)	2.43	(0.68)	+120%	0.1258
IL-11 [pg/ml]	13.0	(4.73)	17.0	(5.59)	+31%	0.5903	0.92	(0.92)	11.2	(2.74)	+1118%	0.0018
LIF [pg/ml]	94.1	(16.9)	72.4	(5.90)	-23%	0.2397	5.53	(2.20)	16.5	(3.34)	+199%	0.0116
OSM [pg/ml]	17.6	(12.4)	4.90	(2.72)	-72%	0.3279	8.30	(3.39)	33.1	(8.12)	+299%	0.0102
**IL-1 family**												
IL-1α [pg/ml]	959	(45.2)	646	(56.6)	-33%	0.0003	298	(47.7)	248	(24.8)	-17%	0.3642
IL-1β [ng/ml]	1.26	(0.06)	1.39	(0.05)	+10%	0.1098	0.05	(0.03)	0.10	(0.04)	+90%	0.3678
IL-18 [pg/ml]	1.43	(0.04)	2.27	(0.20)	+58%	0.0005	0.07	(0.03)	0.18	(0.04)	+162%	0.0193
**Growth factors**												
EGF [pg/ml]	18.2	(2.33)	26.3	(1.75)	+44%	0.0111	8.53	(2.51)	7.62	(2.16)	-11%	0.7841
FGF-9 [ng/ml]	0.23	(0.23)	n/d		-	0.3282	0.60	(0.15)	1.70	(0.28)	+182%	0.0021
FGF-b (FGF-2) [ng/ml]	1.43	(0.05)	1.49	(0.03)	+4%	0.3317	0.77	(0.05)	0.94	(0.06)	+22%	0.0525
Growth Hormone [ng/ml]	18.6	(2.76)	63.6	(9.31)	+241%	0.0001	0.13	(0.13)	0.33	(0.18)	+150%	0.3790
M-CSF (CSF-1) [ng/ml]	3.25	(0.10)	2.98	(0.13)	-8%	0.1200	0.05	(0.01)	0.09	(0.01)	+68%	0.0289
SCF (Kitl) [pg/ml]	75.8	(5.66)	98.2	(5.40)	+29%	0.0092	10.7	(3.28)	24.3	(5.05)	+127%	0.0349
TPO [ng/ml]	16.1	(0.36)	14.9	(0.58)	-7%	0.1024	0.40	(0.18)	2.15	(0.36)	+440%	0.0003
VEGF-A [ng/ml]	0.28	(0.03)	0.18	(0.01)	-36%	0.0051	1.50	(0.22)	1.11	(0.12)	-28%	0.1136

Supplementary table 2) Comparison of disease parameters and analyte concentrations in serum and saliva (continuation)

	Serum						Saliva					
	Strain					*t*-test	Strain					*t*-test
	Balb/c		NOD			(sig. 2-	Balb/c		NOD			(sig. 2-
**Peptide hormones**	Mean	SEM	Mean	SEM	Change	tailed)	Mean	SEM	Mean	SEM	Change	tailed)
Leptin [pg/ml]	643	(141)	607	(113)	-6%	0.8441	n/d		5.40	(2.44)	-	0.0374
Endothelin-1 [pg/ml]	40.0	(7.38)	65.8	(6.92)	+64%	0.0184	5.72	(1.14)	6.93	(1.47)	+21%	0.5224
Insulin [mIU/ml]	2.63	(0.13)	3.88	(0.12)	+48%	< 0.0001	0.61	(0.12)	0.51	(0.10)	-17%	0.5025
**Acute-phase reactants**												
CRP [μg/ml]	1.50	(0.11)	2.77	(0.16)	+85%	< 0.0001	0.03	(0.01)	0.03	(0.01)	-6%	0.8487
Haptoglobin [μg/ml]	18.9	(0.87)	21.6	(0.82)	+14%	0.0359	1.50	(0.32)	2.29	(0.64)	+53%	0.2584
SAP [μg/ml]	17.5	(1.01)	25.0	(0.94)	+43%	< 0.0001	0.13	(0.03)	0.13	(0.03)	+0%	0.9965
SGOT [μg/ml]	15.4	(1.00)	20.4	(1.33)	+33%	0.0062	38.5	(8.45)	40.7	(7.46)	+6%	0.8487
**Coagulation factors**												
Factor III [ng/ml]	1.93	(0.20)	2.78	(0.26)	+45%	0.0173	0.20	(0.05)	0.28	(0.06)	+40%	0.3069
Factor VII [ng/ml]	1.83	(0.27)	2.83	(0.22)	+54%	0.0092	0.38	(0.04)	0.43	(0.04)	+13%	0.3914
Fibrinogen [μg/ml]	34.0	(20.0)	404	(67.3)	+1088%	< 0.0001	318	(87.5)	216	(61.7)	-32%	0.4255
vWF [ng/ml]	34.8	(2.00)	33.3	(2.73)	-4%	0.6616	0.26	(0.06)	0.39	(0.08)	+50%	0.1965
**Adhesion molecules**												
VCAM-1 [ng/ml]	966	(32.6)	1,342	(54.7)	+39%	< 0.0001	0.15	(0.03)	0.22	(0.02)	+49%	0.0424
**Proteases and inhibitors**												
MMP-9 [ng/ml]	73.5	(9.91)	44.4	(5.35)	-40%	0.0170	40.5	(7.02)	69.7	(9.15)	+72%	0.0191
TIMP-1 [ng/ml]	1.22	(0.30)	1.49	(0.10)	+23%	0.3933	0.10	(0.01)	0.16	(0.02)	+58%	0.0444
Cystatin-C [ng/ml]	506	(22.6)	790	(29.0)	+56%	< 0.0001	n/m		n/m		-	-
**Innate immunity**												
MPO [ng/ml]	60.8	(3.06)	102	(8.11)	+67%	0.0001	39.9	(8.43)	78.5	(10.8)	+97%	0.0100
NGAL [ng/ml]	n/d		n/d		-	-	n/m		n/m		-	-
**Miscellaneous**												
Apo A1 [μg/ml]	52.6	(1.55)	63.3	(2.70)	+20%	0.0024	0.48	(0.06)	0.385	(0.08)	-20%	0.3281
B$_2$m [ng/ml]	333	(96.2)	409	(116)	+23%	0.6194	n/m		n/m		-	-
Calbindin [pg/ml]	317	(176)	670	(377)	+111%	0.4046	n/m		n/m		-	-
Clusterin [μg/ml]	202	(5.07)	199	(9.32)	-1%	0.7919	n/m		n/m		-	-
GST-α [pg/ml]	137	(44.4)	252	(214)	+83%	0.6068	12.5	(12.5)	59.1	(40.6)	+373%	0.2678
GST-Mu [ng/ml]	n/d		n/d		-	-	n/m		n/m		-	-
Myoglobin [ng/ml]	824	(229)	1,318	(362)	+60%	0.2607	0.14	(0.04)	0.15	(0.03)	+14%	0.7143
IgA [μg/ml]	137	(5.85)	64.9	(2.96)	-53%	< 0.0001	9.13	(2.72)	9.68	(1.52)	+6%	0.8701
**Autoantibodies**												
anti-Insulin [U]	2.48	(0.34)	5.28	(1.95)	+112%	0.1731	0.71	(0.02)	0.93	(0.12)	+32%	0.0415
anti-M3R IgG [% pos.]	16.0	(0.75)	23.5	(5.71)	+47%	0.2048	n/m		n/m		-	-
anti-M3R IgG1 [% pos.]	0.53	(0.13)	9.77	(2.76)	+1756%	0.0029	n/m		n/m		-	-
anti-M3R IgG2b [% pos.]	1.58	(0.09)	17.7	(4.57)	+1023%	0.0019	n/m		n/m		-	-
anti-M3R IgG2c [% pos.]	0.91	(0.07)	70.5	(7.10)	+7628%	< 0.0001	n/m		n/m		-	-
anti-M3R IgG3 [% pos.]	0.25	(0.07)	0.54	(0.09)	+114%	0.0219	n/m		n/m		-	-
SSA (anti-Ro) [U]	1.03	(0.41)	0.45	(0.26)	-56%	0.2464	-1.2	(0.19)	-0.2	(0.53)	-79%	0.0696
SSB (anti-La) [U]	2.12	(0.26)	1.95	(0.24)	-8%	0.6466	0.71	(0.02)	0.85	(0.06)	+20%	0.0155
anti-RNP [U]	5.68	(0.69)	55.7	(42.1)	+882%	0.2474	0.56	(0.03)	0.83	(0.12)	+46%	0.0174
anti-pCNA [U]	1.70	(0.17)	1.76	(0.24)	+4%	0.8314	0.74	(0.02)	0.81	(0.05)	+10%	0.1278
anti-Proteinase 3 [U]	1.65	(0.17)	1.69	(0.12)	+2%	0.8603	0.64	(0.04)	0.80	(0.09)	+25%	0.0777
anti-Sm [U]	2.58	(0.59)	401	(200)	15432%	0.0586	0.73	(0.02)	2.56	(1.52)	+252%	0.1505
anti-beta 2GPI [U]	8.98	(2.02)	9.21	(2.76)	+3%	0.9463	0.55	(0.03)	0.86	(0.13)	+56%	0.0117
anti-MPO [U]	3.87	(0.55)	3.32	(1.08)	-14%	0.6556	0.36	(0.03)	0.42	(0.06)	+18%	0.3188
anti-Jo1 [U]	0.57	(0.12)	0.64	(0.10)	+13%	0.6374	0.34	(0.05)	0.58	(0.11)	+69%	0.0548
anti-Mitochondrial [U]	3.80	(0.71)	3.47	(0.60)	-9%	0.7217	0.35	(0.04)	0.61	(0.13)	+72%	0.0392
anti-SCL70 [U]	11.6	(2.41)	8.93	(1.41)	-23%	0.3581	0.87	(0.01)	1.22	(0.12)	+40%	0.0021
anti-Ribosomal P [U]	2.15	(0.21)	1.75	(0.17)	-19%	0.1572	0.55	(0.04)	0.66	(0.11)	+19%	0.3113

Salivary flow is expressed as μl saliva secreted per minute normalized per gram bodyweight, focus score is the number of foci with 50 or more mononuclear cells per mm^2 glandular tissue and the ratio index represents the area of inflamed tissue per area of glandular tissue. Standard error of the mean (SEM) is displayed parentheses. Significant differences between the means are filled with red. Abbreviations not introduced in the main manuscript: B2m (beta-2 microglobulin), GST (glutathione S-transferase), Jo-1 (histidyl-RNA synthetase), NGAL (lipocalin-2), pCNA (proliferating cell nuclear antigen), Sm (Smith), RNP (ribonuclear protein), TNF (tumor necrosis factor).

Supplementary table 3) Principal component analyses - component structure and loadings

Serum

Chemokines	1	2	3	
MCP-1 (CCL-2)	0.94	0.24	0.11	
MCP-3 (CCL-7)	0.90	0.38	0.06	
MIP-2 (CXCL-2)	0.84	-0.27	-0.03	
IP-10 (CXCL-10)	0.79	0.24	0.30	
MIP-1α (CCL-3)	0.14	0.87	0.35	
MIP-1γ (CCL-9)	-0.08	0.85	0.09	
MCP-5 (CCL-12)	0.39	0.82	-0.14	
MIP-3β (CCL-19)	-0.19	0.22	0.89	
LTN (XCL-1)	0.47	0.12	0.74	
MDC (CCL-22)	0.58	-0.15	0.66	
Variance explained:	38%	26%	20%	**84%**

Cytokines	1	2	3	4	5	
OPN (SPP-1)	0.94	-0.07	-0.29	0.01	0.03	
LIF	0.74	0.33	0.33	-0.34	-0.23	
IL-1α	0.06	-0.94	0.25	-0.08	-0.02	
CD40L	0.45	0.68	0.03	0.34	0.31	
CD40	-0.11	-0.22	0.95	-0.06	-0.12	
IL-10	-0.09	0.16	-0.06	0.96	-0.04	
IL-18	-0.05	0.10	-0.11	-0.03	0.97	
Variance explained:	24%	22%	17%	17%	16%	**95%**

Growth factors	1	2	3	
VEGF-A	0.97	0.04	-0.09	
SCF (Kitl)	0.93	0.17	0.20	
M-CSF (CSF-1)	-0.06	0.93	0.18	
Growth Hormone	0.34	0.86	0.13	
EGF	0.05	0.21	0.97	
Variance explained:	39%	33%	21%	**93%**

Coagulation factors	1	2	
Factor VII	0.96	-0.10	
Factor III	0.89	0.33	
Fibrinogen	0.07	0.99	
Variance explained:	57%	37%	**94%**

Saliva

CCL & XCL chemokines	1	2	
LTN (XCL-1)	0.98	0.12	
MCP-3 (CCL-7)	0.96	0.17	
MIP-1β (CCL-4)	0.96	0.19	
MCP-1 (CCL-2)	0.94	0.25	
RANTES (CCL-5)	0.67	0.30	
Eotaxin (CCL-11)	0.20	0.91	
MDC (CCL-22)	0.18	0.91	
Variance explained:	60%	27%	**87%**

CXCL chemokines	1	2	
GRO (CXCL-1)	0.92	-0.08	
MIP-2 (CXCL-2)	0.90	0.05	
IP-10 (CXCL-10)	0.78	-0.55	
GCP-2 (CXCL-5)	0.73	0.64	
Variance explained:	48%	39%	**88%**

Cytokines	1	2	3	
IFN-γ	0.98	-0.13	0.05	
IL-7	0.96	0.20	0.14	
OSM	0.94	0.14	0.23	
GM-CSF (CSF-2)	0.89	-0.14	0.40	
IL-17	0.86	-0.03	0.48	
IL-11	0.82	0.39	0.36	
IL-5	0.64	0.28	0.17	
IL-18	0.62	-0.11	0.60	
LIF	0.13	0.98	-0.04	
IL-10	0.12	0.97	0.06	
IL-1β	-0.08	0.88	0.32	
CD40	0.23	0.16	0.96	
CD40L	0.47	0.38	0.77	
Variance explained:	47%	24%	20%	**91%**

Growth factors	1	2	
SCF (Kitl)	0.93	-0.34	
FGF-9	0.90	-0.40	
TPO	0.61	0.55	
M-CSF (CSF-1)	0.46	0.74	
Variance explained:	49%	35%	**84%**

Acute-phase reactants	1	
CRP	0.99	
SAP	0.97	
SGOT	0.97	
Variance explained:	95%	**95%**

Coagulation factors	1	2	
Factor III	0.99	-0.06	
vWF	-0.74	-0.65	
Fibrinogen	-0.01	0.99	
Variance explained:	51%	47%	**98%**

Autoantibodies	1	2	
anti-RNP	0.99	0.01	
anti-Insulin	0.97	0.14	
anti-Mitochondrial	0.96	0.12	
SSB (anti-La)	0.95	0.26	
anti-SCL70	0.86	0.47	
anti-beta 2GPI	0.12	0.99	
Variance explained:	75%	22%	**97%**

Yellow fill indicates significant positive factor loading, green fill significant negative factor loading. The percentage of variances explained (extraction sums of squared loadings) by the component is indicted at the bottom of the column together with cumulative variance explained by all components (bold). In saliva, CCL and XCL and CXCL chemokines were separately investigated in saliva to improve the comprehensibility of the model.

Supplementary table 4) Strain membership prediction by defining variables from principal components

Components serum	R*	Speci-ficity	Sensi-tivity	Hit rate
Growth factor Se-C-1	0.883	100%	92%	96%
Cytokine Se-C-1	0.832	92%	100%	96%
Chemokine Se-C-2	0.821	83%	92%	88%
Growth factor Se-C-2	0.794	75%	92%	83%
Chemokine Se-C-1	0.782	75%	100%	88%
Cytokine Se-C-2	0.774	92%	75%	83%
Chemokine Se-C-3	0.772	92%	83%	88%
Coagulation factor Se-C-2	0.747	83%	100%	92%
Cytokine Se-C-5	0.653	75%	100%	88%
Cytokine Se-C-3	0.645	67%	92%	79%
Coagulation factor Se-C-1	0.532	83%	67%	75%
Growth factor Se-C-3	0.509	75%	67%	71%
Cytokine Se-C-4	0.471	75%	83%	79%
**Components saliva**				
CCL; XCL Sa-C-1	0.880	67%	100%	83%
CXCL Sa-C-1	0.877	92%	100%	96%
Growth factor Sa-C-1	0.799	58%	100%	79%
CXCL Sa-C-2	0.745	75%	92%	83%
Autoantibody Sa-C-1	0.721	63%	100%	85%
Cytokine Sa-C-1	0.678	58%	67%	63%
Cytokine Sa-C-2	0.556	67%	58%	63%
Autoantibody Sa-C-2	0.551	50%	83%	70%
CCL; XCL Sa-C-2	0.539	58%	75%	67%
Cytokine Sa-C-3	0.467	45%	75%	61%
Growth factor Sa-C-2	0.446	58%	67%	63%
Coagulation factor Sa-C-1	0.393	58%	50%	54%
Coagulation factor Sa-C-2	0.281	57%	58%	58%
Acute-phase reac. Sa-C-1	0.205	14%	33%	26%

Results from DA sorted corresponding to their canonical correlation and ranked the principal components according to their capacity to predict strain-membership. Defining variables of the corresponding component were entered simultaneously for DA. Specificity (percentage of correct predictions in the NOD group), sensitivity (percentage of correct predictions in the Balb/c group) and hit ratio (% of correctly classified cases) represent results obtained from cross-validated (leave-one-out) group prediction analyses.

Supplementary figure 1) Extraglandular disease manifestations in the kidneys and the lungs present in a subset of NOD mice

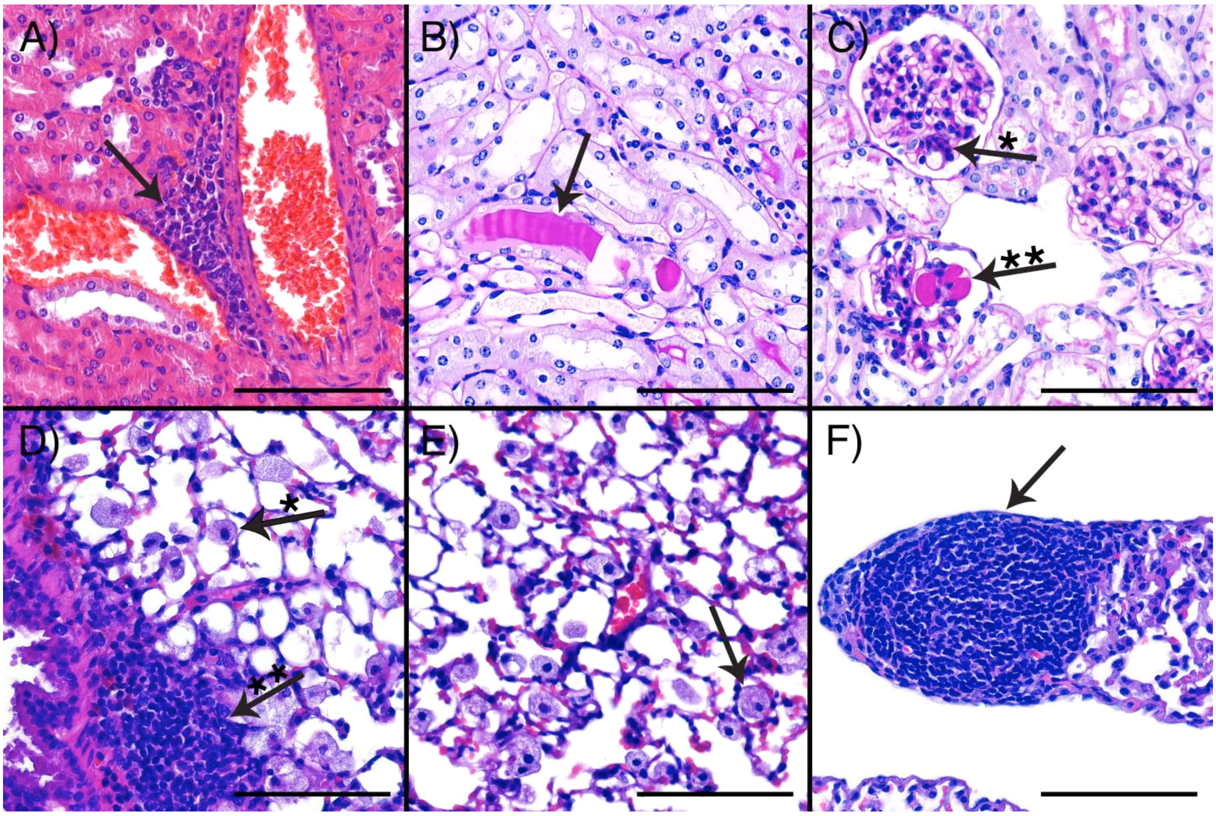

Histopathology of the kidneys (A-C) and the lungs (D-F). A) Mononuclear cell infiltration in the kidney (H&E); B) presence of a hyaline cast in a renal tubulus, (PAS); C) * increased numbers of mesangial cells within the glomerulus, ** hyaline material within glomerular capillaries (PAS); D) * intraalveolar macrophages ** peribronchial lymphocytic infiltrate (H&E); E) foamy macrophages in absence of mononuclear cell infiltration (H&E); F) peripheral lymphocytic infiltrate (H&E). The scale bar represents 100μm; H&E = haematoxylin and eosin; PAS = periodic acid-Schiff staining.

References supplementary table 1)

1. Tornwall J, Lane TE, Fox RI, Fox HS: **T cell attractant chemokine expression initiates lacrimal gland destruction in nonobese diabetic mice**. *Lab Invest* 1999, **79**:1719-1726.
2. Szodoray P, Alex P, Jonsson MV, Knowlton N, Dozmorov I, Nakken B, Delaleu N, Jonsson R, Centola M: **Distinct profiles of Sjögren's syndrome patients with ectopic salivary gland germinal centers revealed by serum cytokines and BAFF**. *Clin Immunol* 2005, **117**:168-176.
3. Szodoray P, Alex P, Brun JG, Centola M, Jonsson R: **Circulating cytokines in primary Sjögren's syndrome determined by a multiplex cytokine array system**. *Scand J Immunol* 2004, **59**:592-599.
4. Cuello C, Palladinetti P, Tedla N, Di Girolamo N, Lloyd AR, McCluskey PJ, Wakefield D: **Chemokine expression and leucocyte infiltration in Sjögren's syndrome**. *Br J Rheumatol* 1998, **37**:779-783.
5. Lodde BM, Mineshiba F, Wang J, Cotrim AP, Afione S, Tak PP, Baum BJ: **Effect of human vasoactive intestinal peptide gene transfer in a murine model of Sjögren's syndrome**. *Ann Rheum Dis* 2006, **65**:195-200.
6. Streckfus C, Bigler L, Navazesh M, Al-Hashimi I: **Cytokine concentrations in stimulated whole saliva among patients with primary Sjögren's syndrome, secondary Sjögren's syndrome, and patients with primary Sjögren's syndrome receiving varying doses of interferon for symptomatic treatment of the condition: a preliminary study**. *Clin Oral Investig* 2001, **5**:133-135.
7. Fox RI, Kang HI, Ando D, Abrams J, Pisa E: **Cytokine mRNA expression in salivary gland biopsies of Sjögren's syndrome**. *J Immunol* 1994, **152**:5532-5539.
8. De Vita S, Dolcetti R, Ferraccioli G, Pivetta B, De Re V, Gloghini A, D'Agosto A, Bartoli E, Carbone A, Boiocchi M: **Local cytokine expression in the progression toward B cell malignancy in Sjögren's syndrome**. *J Rheumatol* 1995, **22**:1674-1680.
9. Jones DT, Monroy D, Ji Z, Atherton SS, Pflugfelder SC: **Sjögren's syndrome: cytokine and Epstein-Barr viral gene expression within the conjunctival epithelium**. *Invest Ophthalmol Vis Sci* 1994, **35**:3493-3504.
10. Kolkowski EC, Reth P, Pelusa F, Bosch J, Pujol-Borrell R, Coll J, Jaraquemada D: **Th1 predominance and perforin expression in minor salivary glands from patients with primary Sjögren's syndrome**. *J Autoimmun* 1999, **13**:155-162.
11. Jonsson MV, Delaleu N, Brokstad KA, Berggreen E, Skarstein K: **Impaired salivary gland function in NOD mice: association with changes in cytokine profile but not with histopathologic changes in the salivary gland**. *Arthritis Rheum* 2006, **54**:2300-2305.
12. Cha S, Brayer J, Gao J, Brown V, Killedar S, Yasunari U, Peck AB: **A dual role for interferon-gamma in the pathogenesis of Sjögren's syndrome-like autoimmune exocrinopathy in the nonobese diabetic mouse**. *Scand J Immunol* 2004, **60**:552-565.
13. Gao J, Killedar S, Cornelius JG, Nguyen C, Cha S, Peck AB: **Sjögren's syndrome in the NOD mouse model is an interleukin-4 time-dependent, antibody isotype-specific autoimmune disease**. *J Autoimmun* 2006, **26**:90-103.
14. Brayer JB, Cha S, Nagashima H, Yasunari U, Lindberg A, Diggs S, Martinez J, Goa J, Humphreys-Beher MG, Peck AB: **IL-4-dependent effector phase in autoimmune exocrinopathy as defined by the NOD.IL-4-gene knockout mouse model of Sjögren's syndrome**. *Scand J Immunol* 2001, **54**:133-140.

15. Kok MR, Yamano S, Lodde BM, Wang J, Couwenhoven RI, Yakar S, Voutetakis A, Leroith D, Schmidt M, Afione S *et al*: **Local adeno-associated virus-mediated interleukin 10 gene transfer has disease-modifying effects in a murine model of Sjögren's syndrome**. *Hum Gene Ther* 2003, **14**:1605-1618.
16. Goules A, Tzioufas AG, Manousakis MN, Kirou KA, Crow MK, Routsias JG: **Elevated levels of soluble CD40 ligand (sCD40L) in serum of patients with systemic autoimmune diseases**. *J Autoimmun* 2006, **26**:165-171.
17. Dimitriou ID, Kapsogeorgou EK, Moutsopoulos HM, Manoussakis MN: **CD40 on salivary gland epithelial cells: high constitutive expression by cultured cells from Sjögren's syndrome patients indicating their intrinsic activation**. *Clin Exp Immunol* 2002, **127**:386-392.
18. Eriksson P, Andersson C, Ekerfelt C, Ernerudh J, Skogh T: **Relationship between serum levels of IL-18 and IgG1 in patients with primary Sjögren's syndrome, rheumatoid arthritis and healthy controls**. *Clin Exp Immunol* 2004, **137**:617-620.
19. Bombardieri M, Barone F, Pittoni V, Alessandri C, Conigliaro P, Blades MC, Priori R, McInnes IB, Valesini G, Pitzalis C: **Increased circulating levels and salivary gland expression of interleukin-18 in patients with Sjögren's syndrome: relationship with autoantibody production and lymphoid organization of the periductal inflammatory infiltrate**. *Arthritis Res Ther* 2004, **6**:R447-456.
20. Koski H, Konttinen YT, Hietanen J, Tervo T, Malmstrom M: **Epidermal growth factor, transforming growth factor-alpha, and epidermal growth factor receptor in labial salivary glands in Sjögren's syndrome**. *J Rheumatol* 1997, **24**:1930-1935.
21. Robinson CP, Yamamoto H, Peck AB, Humphreys-Beher MG: **Genetically programmed development of salivary gland abnormalities in the NOD (nonobese diabetic)-scid mouse in the absence of detectable lymphocytic infiltration: a potential trigger for sialoadenitis of NOD mice**. *Clin Immunol Immunopathol* 1996, **79**:50-59.
22. Boras VV, Cikes N, Lukac J, Cekic-Arambasin A, Virag M, Bosnjak A: **The significance of salivary and serum interleukin 6 and basic fibroblast growth factor levels in patients with Sjögren's syndrome**. *Coll Antropol* 2004, **28 Suppl 2**:305-309.
23. Denko CW, Boja B, Malemud CJ: **Intra-erythrocyte deposition of growth hormone in rheumatic diseases**. *Rheumatol Int* 2003, **23**:11-14.
24. Moutsopoulos HM, Elkon KB, Mavridis AK, Acritidis NC, Hughes GR, Pepys MB: **Serum C-reactive protein in primary Sjögren's syndrome**. *Clin Exp Rheumatol* 1983, **1**:57-58.
25. Youinou P, Fauquert P, Pennec YL, Bendaoud B, Katsikis P, Le Goff P: **Raised C-reactive protein response in rheumatoid arthritis patients with secondary Sjögren's syndrome**. *Rheumatol Int* 1990, **10**:39-41.
26. Saso L, Silvestrini B, Guglielmotti A, Lahita R, Cheng CY: **Abnormal glycosylation of alpha 2-macroglobulin, a non-acute-phase protein in patients with autoimmune diseases**. *Inflammation* 1993, **17**:465-479.
27. Ryo K, Yamada H, Nakagawa Y, Tai Y, Obara K, Inoue H, Mishima K, Saito I: **Possible involvement of oxidative stress in salivary gland of patients with Sjögren's syndrome**. *Pathobiology* 2006, **73**:252-260.
28. Oxholm P, Oxholm A, Manthorpe R: **Epidermal Langerhans cells in patients with primary Sjögren's syndrome**. *Allergy* 1986, **41**:429-434.
29. Blann AD, Hopkins J, Winkles J, Wainwright AC: **Plasma and serum von Willebrand factor antigen concentrations in connective tissue disorders**. *Ann Clin Biochem* 1992, **29 (Pt 1)**:67-71.

30. Cuida M, Halse AK, Johannessen AC, Tynning T, Jonsson R: **Indicators of salivary gland inflammation in primary Sjögren's syndrome**. *Eur J Oral Sci* 1997, **105**:228-233.
31. Turkcapar N, Sak SD, Saatci M, Duman M, Olmez U: **Vasculitis and expression of vascular cell adhesion molecule-1, intercellular adhesion molecule-1, and E-selectin in salivary glands of patients with Sjögren's syndrome**. *J Rheumatol* 2005, **32**:1063-1070.
32. Mikulowska-Mennis A, Xu B, Berberian JM, Michie SA: **Lymphocyte migration to inflamed lacrimal glands is mediated by vascular cell adhesion molecule-1/alpha(4)beta(1) integrin, peripheral node addressin/l-selectin, and lymphocyte function-associated antigen-1 adhesion pathways**. *Am J Pathol* 2001, **159**:671-681.
33. Hulkkonen J, Pertovaara M, Antonen J, Pasternack A, Hurme M, Pollanen P, Lehtimaki T: **Matrix metalloproteinase 9 (MMP-9) gene polymorphism and MMP-9 plasma levels in primary Sjögren's syndrome**. *Rheumatology (Oxford)* 2004, **43**:1476-1479.
34. Azuma M, Aota K, Tamatani T, Motegi K, Yamashita T, Harada K, Hayashi Y, Sato M: **Suppression of tumor necrosis factor alpha-induced matrix metalloproteinase 9 production by the introduction of a super-repressor form of inhibitor of nuclear factor kappaBalpha complementary DNA into immortalized human salivary gland acinar cells. Prevention of the destruction of the acinar structure in Sjögren's syndrome salivary glands**. *Arthritis Rheum* 2000, **43**:1756-1767.
35. Asatsuma M, Ito S, Watanabe M, Takeishi H, Nomura S, Wada Y, Nakano M, Gejyo F, Igarashi A: **Increase in the ratio of matrix metalloproteinase-9 to tissue inhibitor of metalloproteinase-1 in saliva from patients with primary Sjögren's syndrome**. *Clin Chim Acta* 2004, **345**:99-104.
36. Perez P, Kwon YJ, Alliende C, Leyton L, Aguilera S, Molina C, Labra C, Julio M, Leyton C, Gonzalez MJ: **Increased acinar damage of salivary glands of patients with Sjögren's syndrome is paralleled by simultaneous imbalance of matrix metalloproteinase 3/tissue inhibitor of metalloproteinases 1 and matrix metalloproteinase 9/tissue inhibitor of metalloproteinases 1 ratios**. *Arthritis Rheum* 2005, **52**:2751-2760.
37. Yamachika S, Nanni JM, Nguyen KH, Garces L, Lowry JM, Robinson CP, Brayer J, Oxford GE, da Silveira A, Kerr M *et al*: **Excessive synthesis of matrix metalloproteinases in exocrine tissues of NOD mouse models for Sjögren's syndrome**. *J Rheumatol* 1998, **25**:2371-2380.
38. Cha S, van Blockland SC, Versnel MA, Homo-Delarche F, Nagashima H, Brayer J, Peck AB, Humphreys-Beher MG: **Abnormal organogenesis in salivary gland development may initiate adult onset of autoimmune exocrinopathy**. *Exp Clin Immunogenet* 2001, **18**:143-160.
39. Ryu OH, Atkinson JC, Hoehn GT, Illei GG, Hart TC: **Identification of parotid salivary biomarkers in Sjögren's syndrome by surface-enhanced laser desorption/ionization time-of-flight mass spectrometry and two-dimensional difference gel electrophoresis**. *Rheumatology (Oxford)* 2006, **45**:1077-1086.
40. Ludviksdottir D, Janson C, Hogman M, Gudbjornsson B, Bjornsson E, Valtysdottir S, Hedenstrom H, Venge P, Boman G: **Increased nitric oxide in expired air in patients with Sjögren's syndrome. BHR study group. Bronchial hyperresponsiveness**. *Eur Respir J* 1999, **13**:739-743.
41. Navone R, Lunardi C, Gerli R, Tinazzi E, Peterlana D, Bason C, Corrocher R, Puccetti A: **Identification of tear lipocalin as a novel autoantigen target in Sjögren's syndrome**. *J Autoimmun* 2005, **25**:229-234.

42. Lodde BM, Sankar V, Kok MR, Leakan RA, Tak PP, Pillemer SR: **Serum lipid levels in Sjögren's syndrome**. *Rheumatology (Oxford)* 2006, **45**:481-484.
43. Vaudo G, Bocci EB, Shoenfeld Y, Schillaci G, Wu R, Del Papa N, Vitali C, Delle Monache F, Marchesi S, Mannarino E *et al*: **Precocious intima-media thickening in patients with primary Sjögren's syndrome**. *Arthritis Rheum* 2005, **52**:3890-3897.
44. Gottenberg JE, Busson M, Cohen-Solal J, Lavie F, Abbed K, Kimberly RP, Sibilia J, Mariette X: **Correlation of serum B lymphocyte stimulator and beta2 microglobulin with autoantibody secretion and systemic involvement in primary Sjögren's syndrome**. *Ann Rheum Dis* 2005, **64**:1050-1055.
45. Markusse HM, Huysen JC, Nieuwenhuys EJ, Swaak AJ: **Beta 2 microglobulin in tear fluid from patients with primary Sjögren's syndrome**. *Ann Rheum Dis* 1992, **51**:503-505.
46. Morinobu A, Kanagawa S, Koshiba M, Sugai S, Kumagai S: **Association of the glutathione S-transferase M1 homozygous null genotype with susceptibility to Sjögren's syndrome in Japanese individuals**. *Arthritis Rheum* 1999, **42**:2612-2615.
47. Bendaoud B, Pennec YL, Lelong A, Le Noac'h JF, Magadur G, Jouquan J, Youinou P: **IgA-containing immune complexes in the circulation of patients with primary Sjögren's syndrome**. *J Autoimmun* 1991, **4**:177-184.
48. Rhodus N, Dahmer L, Lindemann K, Rudney J, Mathur A, Bereuter J: **s-IgA and cytokine levels in whole saliva of Sjögren's syndrome patients before and after oral pilocarpine hydrochloride administration: a pilot study**. *Clin Oral Investig* 1998, **2**:191-196.
49. Zandbelt MM, Wentink JR, de Wilde PC, van Damme PA, van de Putte LB, van den Hoogen FH: **The synergistic value of focus score and IgA% score of sublabial salivary gland biopsy for the accuracy of the diagnosis of Sjögren's syndrome: a 10-year comparison**. *Rheumatology (Oxford)* 2002, **41**:819-823.
50. Matsumoto N, Salam MA, Watanabe H, Amagasa T, Senpuku H: **Role of gene E2f1 in susceptibility to bacterial adherence of oral streptococci to tooth surfaces in mice**. *Oral Microbiol Immunol* 2004, **19**:270-276.
51. Dawson LJ, Stanbury J, Venn N, Hasdimir B, Rogers SN, Smith PM: **Antimuscarinic antibodies in primary Sjögren's syndrome reversibly inhibit the mechanism of fluid secretion by human submandibular salivary acinar cells**. *Arthritis Rheum* 2006, **54**:1165-1173.
52. Gao J, Cha S, Jonsson R, Opalko J, Peck AB: **Detection of anti-type 3 muscarinic acetylcholine receptor autoantibodies in the sera of Sjögren's syndrome patients by use of a transfected cell line assay**. *Arthritis Rheum* 2004, **50**:2615-2621.
53. Cavill D, Waterman SA, Gordon TP: **Antibodies raised against the second extracellular loop of the human muscarinic M3 receptor mimic functional autoantibodies in Sjögren's syndrome**. *Scand J Immunol* 2004, **59**:261-266.
54. Avaniss-Aghajani E, Berzon S, Sarkissian A: **Clinical value of multiplexed bead-based immunoassays for detection of autoantibodies to nuclear antigens**. *Clin Vaccine Immunol* 2007, **14**:505-509.
55. Halse AK, Marthinussen MC, Wahren-Herlenius M, Jonsson R: **Isotype distribution of anti-Ro/SS-A and anti-La/SS-B antibodies in plasma and saliva of patients with Sjögren's syndrome**. *Scand J Rheumatol* 2000, **29**:13-19.
56. Tengner P, Halse AK, Haga HJ, Jonsson R, Wahren-Herlenius M: **Detection of anti-Ro/SSA and anti-La/SSB autoantibody-producing cells in salivary glands from patients with Sjögren's syndrome**. *Arthritis Rheum* 1998, **41**:2238-2248.
57. Skarstein K, Holmdahl R, Johannessen AC, Goldschmidt T, Jonsson R: **Short-term administration of selected anti-T-cell receptor V beta chain specific MoAb reduces sialadenitis in MRL/lpr mice**. *Scand J Immunol* 1995, **42**:529-534.

58. Ramos-Casals M, Nardi N, Brito-Zeron P, Aguilo S, Gil V, Delgado G, Bove A, Font J: **Atypical autoantibodies in patients with primary Sjögren syndrome: clinical characteristics and follow-up of 82 cases**. *Semin Arthritis Rheum* 2006, **35**:312-321.
59. Tzang BS, Chen TY, Hsu TC, Liu YC, Tsay GJ: **Presentation of autoantibody to proliferating cell nuclear antigen in patients with chronic hepatitis B and C virus infection**. *Ann Rheum Dis* 1999, **58**:630-634.
60. Font J, Ramos-Casals M, Cervera R, Bosch X, Mirapeix E, Garcia-Carrasco M, Morla RM, Ingelmo M: **Antineutrophil cytoplasmic antibodies in primary Sjögren's syndrome: prevalence and clinical significance**. *Br J Rheumatol* 1998, **37**:1287-1291.
61. Cervera R, Garcia-Carrasco M, Font J, Ramos M, Reverter JC, Munoz FJ, Miret C, Espinosa G, Ingelmo M: **Antiphospholipid antibodies in primary Sjögren's syndrome: prevalence and clinical significance in a series of 80 patients**. *Clin Exp Rheumatol* 1997, **15**:361-365.
62. Ikuno N, Mackay IR, Jois J, Omagari K, Rowley MJ: **Antimitochondrial autoantibodies in saliva and sera from patients with primary biliary cirrhosis**. *J Gastroenterol Hepatol* 2001, **16**:1390-1394.
63. Ramos-Casals M, Pares A, Jara LJ, Solans R, Vinas O, Vazquez P, Sanchez-Tapias JM, Rodes J, Font J: **Antimitochondrial antibodies in patients with chronic hepatitis C virus infection: description of 18 cases and review of the literature**. *J Viral Hepat* 2005, **12**:648-654.
64. Goules A, Masouridi S, Tzioufas AG, Ioannidis JP, Skopouli FN, Moutsopoulos HM: **Clinically significant and biopsy-documented renal involvement in primary Sjögren syndrome**. *Medicine (Baltimore)* 2000, **79**:241-249.
